# E2 variants for probing E3 ubiquitin ligase activities

**DOI:** 10.1073/pnas.2524899122

**Published:** 2026-01-02

**Authors:** Jiale Du, Gisele A. Andree, Daniel Horn-Ghetko, Luca Stier, Jaspal Singh, Sebastian Kostrhon, Leo Kiss, Matthias Mann, Sachdev S. Sidhu, Brenda A. Schulman

**Affiliations:** ^a^Department of Molecular Machines and Signaling, Max Planck Institute of Biochemistry, Martinsried 82152, Germany; ^b^Department of Chemistry, School of Natural Sciences, Technical University of Munich, Garching 85748, Germany; ^c^School of Pharmacy, University of Waterloo, Waterloo, ON N2L 3G1, Canada; ^d^Department of Proteomics and Signal Transduction, Max Planck Institute of Biochemistry, Martinsried 82152, Germany

**Keywords:** RBR, E3 ligase, E2, ubiquitin, activity-based probe

## Abstract

E3 ligases partner with E2 enzymes to regulate vast eukaryotic biology. The hierarchical nature of these pairings, with >600 E3s and ~40 E2s in humans, necessitates that E2s cofunction with numerous different E3s. Here, focusing on E3s in the RING-between-RING (RBR) family and their partner UBE2L3 and UBE2D-family E2s, we report an approach to interrogate selected pathways. We screened phage-displayed libraries of structure-based E2 variants (E2Vs) to discover enzymes with enhanced affinity and specificity toward half of all RBR E3 ligases (ARIH1, ARIH2, ANKIB1, CUL9, HOIL1, HOIP, and RNF14). Collectively, these E2Vs allowed distinguishing actions of different cofunctioning E3s, obtaining high-resolution cryogenic Electron Microscopy (cryo-EM) structures of an RBR E3 in the context of a substrate-bound multiprotein complex, and profiling an endogenous RBR E3 response to an extracellular stimulus. Overall, we anticipate that E2V technology will be a generalizable tool to enable in-depth mechanistic and structural analysis of E3 ligase functions, and mapping their activity states and protein partners in cellular signaling cascades.

## Significance

E2 ubiquitin conjugating enzymes and E3 ligases regulate virtually all aspects of eukaryotic cell biology. Despite progress in interrogating functions of these enzymes, understanding their regulation remains challenging due to a paucity of probes for E3 enzymes, and because of E2 promiscuity, since each E2 functions with many E3s. Also, E2–E3 interactions are typically of moderate affinity. Here, focusing on UBE2L3 and UBE2D3 E2s and RING-between-RING (RBR)-type E3s, we present a generalizable approach for engineering E2 variants (E2Vs) with enhanced selectivity and affinity toward particular E3 ligases. Engineered E2Vs allow dissecting E3 functions in a multi-E3 complex, determining E2–E3 complex structures, and probing E3s responding to cellular signals.

Specificity of the ubiquitin (Ub) system depends on complexes between E2 conjugating and E3 ligase enzymes. E2s first receive Ub on their active site cysteine from an E1 enzyme and then bind a cognate E3 that recruits specific substrates for ubiquitylation. In humans, more than 30 different E2 and 600 different E3 enzymes achieve ubiquitylation by various mechanisms (1–3). E3s in the RING and U-box families lack an active site and employ E2 enzymes to transfer Ub to their recruited substrates. Meanwhile, other E3s, such as those in the HECT, RING-Cys-Relay (RCR), and RING-Between-RING (RBR) families, have a catalytic Cys, which receives Ub from an E2 and subsequently transfers this Ub to a substrate (2–5). Collectively, these E2s and E3s control the interactions, subcellular localizations, and half-lives of most intracellular proteins, thereby regulating a vast array of biological processes.

In humans, a family of 14 E3 ligases contain an RBR catalytic domain that functions through a distinctive ubiquitylation mechanism (5–9). A “RING1 domain” binds an E2 in a manner similar to canonical RING or U-box domains. The E2’s thioester-linked Ub is positioned by numerous interactions, including with an “In-Between-RING” (IBR) domain, for transfer to the catalytic Cys in the Rcat (for “Required for catalysis,” also known as RING2) domain (10). Ub is subsequently transferred from the Rcat domain to substrates.

Although isolated RBR domains from some E3s are competent to receive and transfer Ub, many are autoinhibited in the context of their native assemblies (11). Autoinhibition is typically mediated by intramolecular interactions occluding the catalytic Cys in the Rcat domain, for example, in the ARIH family (ARIH1, ARIH2, and CUL9), and restraining E2~Ub-binding domains in inactive arrangements, for example, in ARIH1, PARKIN, HOIP, and other RBR E3s (12–18). In some cases, E3 ligase activation requires posttranslational modification or binding to other proteins that trigger release of autoinhibitory interactions. A well-studied example is release from autoinhibition and activation of the Parkinson’s Disease associated E3, PARKIN, achieved by phosphorylation of its own Ub-like domain and binding phosphorylated Ub (19–23). Meanwhile, other RBR ligases are regulated by forming complexes with other E3s. For example, autoinhibition of ARIH1 and ARIH2 is released when these RBR E3s bind to cognate NEDD8-modified cullin-RING E3 ligases (CRLs). ARIH1 and ARIH2 mediate widespread regulation by ubiquitylating substrates recruited to substrate receptors of CRL E3s (18, 24–26). CUL9 forms a dedicated complex with RBX1, displays neddylated CRL E3 and RBR E3 features in a single polypeptide, and maintains genome integrity (27–30). Meanwhile, dual RBR E3s, HOIL1 and HOIP, cofunction together with the protein SHARPIN in a Linear Ub chain Assembly E3–E3 Complex (LUBAC) that regulates many immune and signaling pathways (31–34). RNF14 triggers the degradation of damaged RNA–protein crosslinks (RPCs) that stall ribosomes by ubiquitylating the stalled complex with atypical K6-linked Ub chains (35, 36). RNF216, which is mutated in Gordon Holmes syndrome, generates K63-linked Ub chains and is allosterically activated by K63-linked di-Ub (37). While little is known about ANKIB1, a recent study showed that it plays a role in immune response by modifying mitochondrial antiviral signaling proteins with K48 chains (38).

While many RBR E3s modify protein lysines, others have the capacity to ubiquitylate noncanonical reactive groups on macromolecules. For example, HOIP catalyzes peptide bond formation between Ub’s C-terminus to Ub’s N terminus, generating “linear” or “M1”-linked poly-Ub chains (31). Interestingly, E3 ligase activity of HOIL1 and HOIP individually, and within LUBAC, is activated through interactions with linear Ub chains (39). Moreover, HOIL1 and ARIH1 have been shown to forge linkages between Ub’s C-terminus and hydroxyls, to achieve noncanonical ubiquitylation on protein serines or threonines and oligosaccharides (40–44).

Due to their biological importance and distinctive features, there is great interest in developing generalizable strategies that can probe entire families of E3 ligases. Several probes marshal the relatively high-affinity E3 interactions with E2~Ub intermediates, where Ub is normally covalently linked to the E2 catalytic Cys. RING and U-box domains can react with a photoactivatable side-chain in Ub that is stably linked to an E2 active site, mimicking an E2~Ub conjugate (45). Meanwhile, probes with electrophiles installed between the E2 and Ub can covalently capture the catalytic Cys of HECT, RBR, and some divergent E3s in a manner mimicking intermediates along Ub transfer from E2 to E3 (4, 46–57). It has also been demonstrated that reactive groups can capture E2 and E3 active sites installed between substrates and ubiquitin (47, 58, 59). Such probes have allowed defining enzyme reaction mechanisms, obtaining high-resolution structures, and tracing pathways and identifying regulation when delivered inside cells. In yet another type of probe, we previously developed “Ub variant” (UbV) probes that have allowed activating, inhibiting, and structurally characterizing otherwise intractable E3 ligases in the RING, U-box, HECT families, and other components of the Ub system, as well as the RBR E3 PARKIN (60–64). The underlying concept is that sequence variants of Ub can bind distinct interactors with higher affinity and selectivity than Ub itself (65). Unlike Ub, UbV sequences are not evolutionarily constrained by necessity to interact with hundreds of protein partners. Thus, in vitro evolution allows discovering UbVs with novel sequences enhancing contacts with E3s.

Much like Ub, the natural sequences of E2 enzymes also must allow binding to various partner E3 ligases (66). In particular, the E2 enzyme UBE2L3 serves as the intermediary in numerous E1–E2–E3 cascades with HECT and RBR-family ligases (5). UBE2D3 is even more promiscuous, with a single surface that interacts with members of all well-characterized families of E3 ligases (67). We thus considered the possibility that alternative sequences at this surface could show enhanced affinity and specificity for particular E3 ligases. In this study, we report in vitro evolution of E2 scaffolds, and use of phage display to select E2 variant (E2V) proteins that bind to specific members of the RBR family. Despite the strong similarity between E2–E3 interactions across families, it was possible to obtain E2Vs with enhanced selectivity, for example, preferentially binding the ARIH-family relative to other RBR E3s, or preferentially functioning with HOIL1 or HOIP enabling separating functions of these E3s within LUBAC. Incorporating E2Vs into activity-based probes (ABPs) expands capacity for proteomic profiling of suites of human E3 ligases.

## Results

### Development of E2 Scaffold Variants to Target RBR E3s.

As a first step toward generating phage-displayed libraries of E2Vs, we assessed the display of human UBE2L3 and UBE2D3 on the phage surface when expressed from a P3 phagemid vector (68). To circumvent any challenges that might arise from reactivity at the active site, the catalytic cysteines were mutated to alanine. Phage enzyme-linked immunosorbent assays (ELISAs) showed that both E2s were displayed at levels approaching that of Ub, which we and others have used extensively as a scaffold for generating protein variants with altered specificity (*SI Appendix*, Fig. S1*A*).

To select residues for varying in E2V libraries, we first inspected published structures showing interactions between E2s and RBR E3s (30, 42, 47, 69–71) (*SI Appendix*, Fig. S1 *B* and *C*). The E2s in these complexes are UBE2L3, which functions in concert with a large cohort of RBR E3s, or a member of the even more promiscuous UBE2D-family. As described previously (69), three E2 regions contact RING1 from all studied RBR E3s. Region 1 corresponds to the E2 N-terminal helix, and Regions 2 and 3 are so-called “Loop 1” and “Loop 2,” named in the original discovery of their mediating interactions with canonical RING and HECT E3s (72, 73). Together, these three regions straddle the zinc-binding loops from RING1 of RBR E3s (*SI Appendix*, Fig. S1*B*). Next, we examined the sequences of all E2s and considered that mutating conserved residues could allow selecting variants that are substantially divergent, and ideally more specific in E3 recognition (*SI Appendix*, Fig. S1*D*). Thus, conserved E2 surface residues in the three RBR RING1-binding regions were selected for randomization in the context of UBE2L3 and UBE2D3 (*SI Appendix*, Fig. S1*E*). Libraries of E2V sequences containing mutations in one of the three regions were constructed for phage display, with theoretical diversity of 6.7 × 10^7^ for the UBE2L3 scaffold, and 1.28 × 10^9^ for the UBE2D3 scaffold.

We also generated a collection of RBR E3 proteins, which we could express in active form without requiring additional modifications, to use as baits for E2V selections. We purified full-length, point mutant, or truncated versions of eight family members (ARIH1, ARIH2, ANKIB1, RNF14, RNF216, CUL9, HOIL1, and HOIP). Constructs were based on previous studies reporting versions that are active for autoubiquitylation (30, 36, 47, 70, 74, 75) (*SI Appendix*, *Supplemental Methods*). For ARIH1 and ARIH2, we used the “open” mutant relieved from autoinhibition (F430A/E431A/E503A in ARIH1 and L381A/E382A/E455A in ARIH2, hereafter referred to as ARIH1^ON^ and ARIH2^ON^ for experiments with recombinant proteins).

**Fig. 1.**
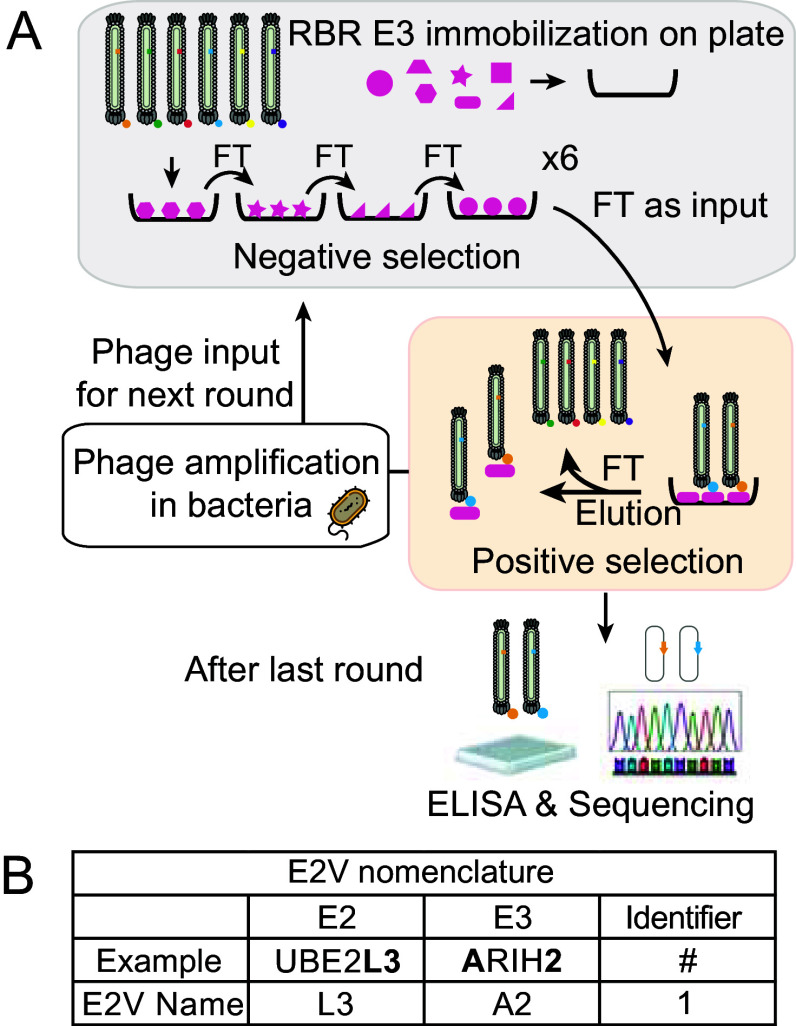
Development of E2Vs for RBR E3 ligases. (*A*) Scheme of phage-displayed library and iterative selection against a panel of RBR E3 ligases. (*B*) Unique nomenclature assigned to E2Vs.

To discover binders with increased specificity for particular E3s, the libraries of phage-displayed E2Vs were subjected to a negative-positive-negative-negative selection protocol (Fig. 1*A*). Positive selection was performed with one RBR E3. Negative selections were performed sequentially with ARIH1^ON^, ARIH2^ON^, ANKIB1, RNF14, RNF216, CUL9-RBX1, HOIL1, and HOIP. For LUBAC, the counter selections were only performed with HOIP and HOIL1 on their own in order to select for E2V binding to LUBAC as a complex in contrast to binding individually to HOIL1 or HOIP. We ultimately purified 23 RBR-targeting E2Vs: three for ARIH1^ON^, two each for ANKIB1, RNF14, CUL9-RBX1, HOIL1, HOIP, four for LUBAC, and six for ARIH2^ON^ (selection for RNF216 did not yield E2Vs with higher affinity compared to WT E2s). We refer to these E2Vs with a three-part nomenclature: 1) L3 or D3, referring to the parental E2 from which the variant is derived; 2) a term for the E3 used for positive selection (A1 for ARIH1, A2 for ARIH2, C9 for CUL9, AN for ANKIB1, R14 for RNF14, HL for HOIL1, HP for HOIP, and LU for LUBAC); and 3) a unique number identifier, based on the order the colony was originally picked (Fig. 1*B*).

Comparing the E2V sequences showed an interesting trend (*SI Appendix*, Fig. S1*F*). Where multiple variants were selected for a given RBR E3, the sequence changes in a particular scaffold typically mapped to a common region. For example, the E2Vs based on the UBE2L3 scaffold that bound ARIH1^ON^, ARIH2^ON^, and ANKIB1 showed variations in Region 1. However, Regions 2 and/or 3 varied in the majority of E2Vs binding LUBAC, its constituent E3s HOIP or HOIL1, and RNF14.

### E2Vs Selected for Binding ARIH1^ON^, CUL9-RBX1, ANKIB1, and RNF14 Showed Modest Increases in Relative Selectivity and Ubiquitylation Activity.

The E2Vs positively selected with ARIH1^ON^, CUL9-RBX1, ANKIB1, and RNF14 were examined in three assays. First, the E2Vs were qualitatively examined for selectivity, by comparing binding across a panel of RBR E3s (these four ligases, and also ARIH2^ON^, RNF216, and LUBAC) to their WT E2 counterparts. Briefly, C-terminally biotinylated E2Vs were immobilized on magnetic streptavidin beads, a purified E3 was added, and interaction was assessed based on pulldown after washing. Second, the E2Vs were assayed for ubiquitylation activity, examined in a pulse–chase format starting with a preformed E2~ or E2V~Ub thioester intermediate. Ub was fluorescently labeled and tracked by SDS-PAGE upon addition of E3. Third, isothermal titration calorimetry (ITC) was used to quantify interactions between the E2V (and a stable mimic of the E2V~Ub thioester intermediate) and the E3 it was selected to bind.

**Fig. 2.**
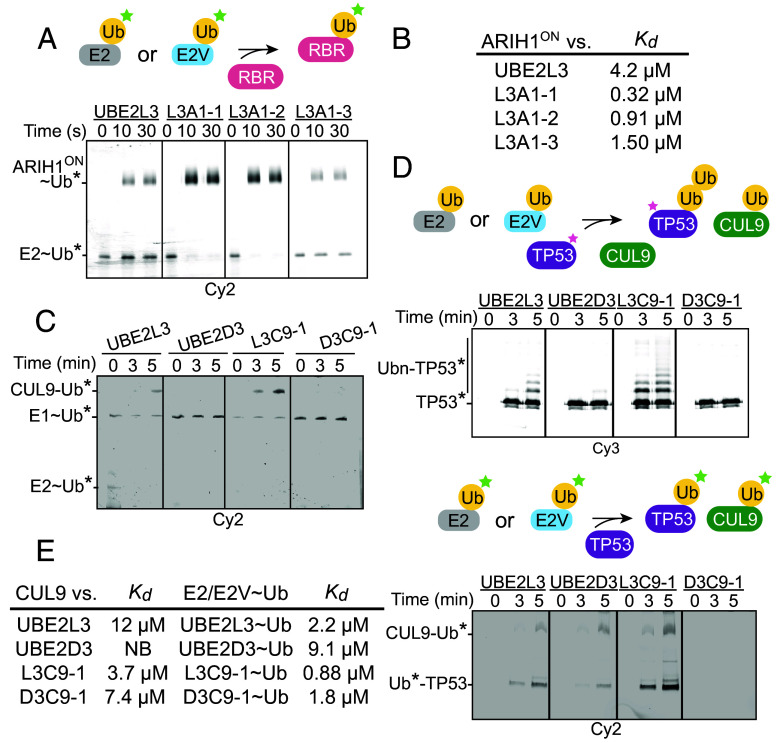
E2Vs for ARIH1^ON^ and CUL9-RBX1 improve the selectivity and ubiquitylation activity. (*A*) Fluorescent Ub (Ub*) transfer from UBE2L3 or E2Vs to ARIH1^ON^. Ub* is detected on nonreducing SDS-PAGE (n = 2 technically independent experiments). (*B*) Dissociation constants (K_d_) between ARIH1^ON^ and UBE2L3 or indicated E2Vs measured by ITC. (*C*) Multiturnover ubiquitylation assay of fluorescent Ub (Ub*), E1, E2, or E2V and CUL9-RBX1 (n = 2 technically independent experiments). (*D*) Assays examining capacity of the indicated E2Vs to support CUL9-RBX1-mediated polyubiquitylation of TP53, monitoring either fluorescently labeled TP53 (TP53*, *Left*) or fluorescent Ub (Ub*, *Right*) (n = 2 technically independent experiments). (*E*) Dissociation constants (K_d_) between CUL9-RBX1 and UBE2L3, UBE2D3, or CUL9-RBX1 E2Vs measured by ITC.

Of the three E2Vs identified for ARIH1^ON^, which all have the UBE2L3 scaffold, two (L3A1-1 and L3A1-2) showed substantially increased selectivity compared to the WT E2 counterpart, losing interaction with ANKIB1, RNF14, RNF216, and CUL9-RBX1 in the pulldown assay (*SI Appendix*, Fig. S2*A*). Notably, the improvements in selectivity were also reflected by greater autoubiquitylation activity toward and affinity for ARIH1^ON^ (Fig. 2*A*). L3A1 showed more than 10-fold higher affinity for ARIH1^ON^ than WT UBE2L3 as measured by ITC (Fig. 2*B*).

Pulldown experiments with the two CUL9-RBX1-selected E2Vs showed altered RBR E3 selectivity compared to the WT E2s. L3C9-1 lost interaction with RNF14 and RNF216, while D3C9-1 gained interaction with CUL9-RBX1 and also with the other ARIH-family RBR E3s ARIH1^ON^ and ARIH2^ON^ (*SI Appendix*, Fig. S2*B*). Meanwhile, L3C9-1 showed superior ubiquitylation of both CUL9-RBX1, and TP53 (Fig. 2 *C* and *D*). This E2V on its own also showed fourfold greater affinity for CUL9-RBX1 (Fig. 2*E*). Both L3C9-1 and D3C9-1 showed improved binding in the context of E2V~Ub complexes.

For ANKIB1, the two selected E2Vs based on the UBE2L3 scaffold showed improved selectivity, losing interaction with RNF14, RNF216, and CUL9-RBX1 (*SI Appendix*, Fig. S2*C*). These both also showed improved capacity to mediate ubiquitylation and binding with ANKIB1 (*SI Appendix*, Fig. S2 *D* and *E*). Meanwhile, the two E2Vs selected for binding RNF14 also showed improved selectivity, losing interaction with ANKIB1, RNF216, and CUL9-RBX1. Additionally, L3R14-2 qualitatively showed reduced binding to ARIH2^ON^ (*SI Appendix*, Fig. S2*F*). Compared to UBE2L3, L3R14-1, and L3R14-2 showed nearly 30-fold and sevenfold improved affinity for RNF14 (*SI Appendix*, Fig. S2*D*).

UBE2L3 is not a preferred E2 for RNF14 (36). Accordingly, this E2 and its derived E2Vs showed little transfer of Ub to RNF14 compared to its preferred partners UBE2D3 and UBE2E1 (*SI Appendix*, Fig. S2*G*). Lack of activity was not apparently due to inability of the E2Vs to receive Ub from E1 (*SI Appendix*, Fig. S2*H*). This assay provided an opportunity for querying a functional effect of E2V–RNF14 interactions, because E1 and E3 binding sites on E2s overlap. Adding catalytically inactive RNF14 to the reactions slowed Ub conjugation to the E2Vs but had little effect on formation of the UBE2L3~Ub conjugate (*SI Appendix*, Fig. S2*I*). Thus, the high-affinity E2–E3 interactions may not translate to superior ligation activity for a noncognate pairing, but may provide opportunities for developing noncovalent inhibitors. Indeed, the catalytic Cys-to-Ala mutants of both E2Vs served as inhibitors when added *in trans* to reactions with WT E2s (*SI Appendix*, Fig. S2 *J* and *K*).

It is interesting that UBE2L3-derived E2Vs showed improved affinity toward RNF14, but failed to rescue the catalytic inactivity of WT UBE2L3. The catalytic defects of UBE2L3 toward RNF14 could suggest structural incompatibility limiting catalysis. Notably, structural models of RNF14–E2 complexes generated by AlphaFold3 showed clashing between the E3 and UBE2L3 catalytic cysteines (*SI Appendix*, Fig. S2*L*). Such clashing was not observed for models with UBE2D3 or UBE2E1 which can function with RNF14.

### Deconvoluting HOIL1 and HOIP Activity within LUBAC Using E3-Specific E2Vs.

**Fig. 3.**
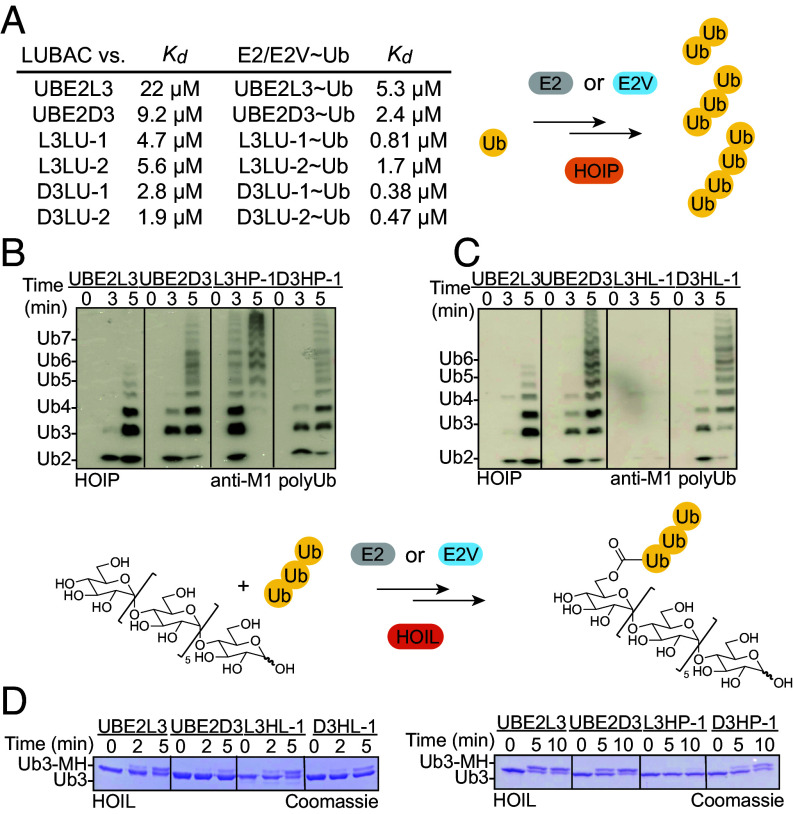
E3-selective E2Vs for HOIL1 and HOIP within LUBAC. (*A*) Dissociation constants (K_d_) between LUBAC and UBE2L3, UBE2D3, or LUBAC-enriched E2Vs measured by ITC. (*B*) Ubiquitin chain formation by HOIP with UBE2L3, UBE2D3, or E2Vs selected for binding HOIP (n = 2 technically independent experiments). (*C*) Same as *B*, except with E2Vs selected for binding HOIL. (*D*) MH ubiquitylation by HOIL1 with WT UBE2L3, UBE2D3, or indicated E2Vs. Reactions were performed with preassembled linear tri-Ub as the source of donor Ub (n = 2 technically independent experiments).

Eight E2Vs selected to bind LUBAC, or its RBR E3 subunits HOIL1 or HOIP (L3HP-1, D3HP-1, L3HL-1, D3HL-1, L3LU-1, L3LU-2, D3LU-1, and D3LU-2) showed improved interactions compared to their WT counterpart E2s (*SI Appendix*, Fig. S3 *A* and *B*). Furthermore, all those in the UBE2L3 scaffold showed improved selectivity through loss of interactions with RNF14 and RNF216. Two (L3HL-1 and L3LU-1) further showed reduced binding to CUL9-RBX1, with the former also losing interaction with ANKIB1 as well (*SI Appendix*, Fig. S3*A*). The four E2Vs selected for binding LUBAC also showed improved affinity, as measured by ITC, compared to their WT counterparts (Fig. 3*A*).

To characterize the E2Vs targeting HOIL1 and HOIP, we established in vitro assays for their individual ubiquitylation activities. HOIP activity was monitored by detecting formation of M1-linked linear Ub chains (*SI Appendix*, Fig. S3*C*). HOIL1 reactions were performed using the oligosaccharide maltoheptaose (MH) as the substrate. HOIL1 is stimulated by the linear chains generated by HOIP (42). Thus, HOIL1 activity was tested with various sources of Ub, ranging from mono Ub to unanchored linear chains of various lengths, where a shift in mobility reflects linkage to MH. HOIL1 activity was greater with longer chains as the sole source of Ub, perhaps due to their promoting formation of the active E3 conformation (*SI Appendix*, Fig. S3*C*). Based on ease of product detection, we used tri-Ub as the source of Ub for subsequent assays of HOIL1 activity.

We then used these assays to interrogate effects of adding the E2Vs. All four E2Vs enriched from selection with LUBAC retained both activities with LUBAC, and activity with the individual E3 subunits HOIL1 and HOIP on their own (*SI Appendix*, Fig. S3 *D* and *E*). Meanwhile, the E2Vs selected to bind HOIP either maintained (D3HP-1) or improved (L3HP-1) its polyubiquitylation activity, while those selected to bind HOIL1 showed lower activity with HOIP compared to their WT E2 counterparts (Fig. 3 *B* and *C*). The E2Vs selected to bind HOIL1 correspondingly maintained activity toward MH, while one selected to bind HOIP (L3HP-1) lost this activity relative to WT UBE2L3 (Fig. 3*D*). The capacity for E2Vs L3HL-1 and L3HP-1 to separate HOIL1 and HOIP functions was also observed when the ubiquitylation assays were performed with both E3s together in LUBAC (*SI Appendix*, Fig. S3 *D* and *F*).

### E2Vs Show Improved Affinity for ARIH2^ON^.

Phage display selection yielded six E2Vs with improved selectivity to ARIH2^ON^ (five derived from the UBE2L3 scaffold and one from the UBE2D3 scaffold). Two, L3A2-2 and L3A2-3, are most selective, binding only ARIH1^ON^ as well as ARIH2^ON^ in our survey with selected purified E3s. Meanwhile, L3A2-1, L3A2-4, and L3A2-5 retained binding toward ARIH1^ON^, ARIH2^ON^, and ANKIB1, and lost interaction with RNF14, RNF216, and CUL9-RBX1. Interestingly, the UBE2D3-derived D3A2-1, also binds LUBAC in addition to ARIH2^ON^ (*SI Appendix*, Fig. S4*A*).

**Fig. 4.**
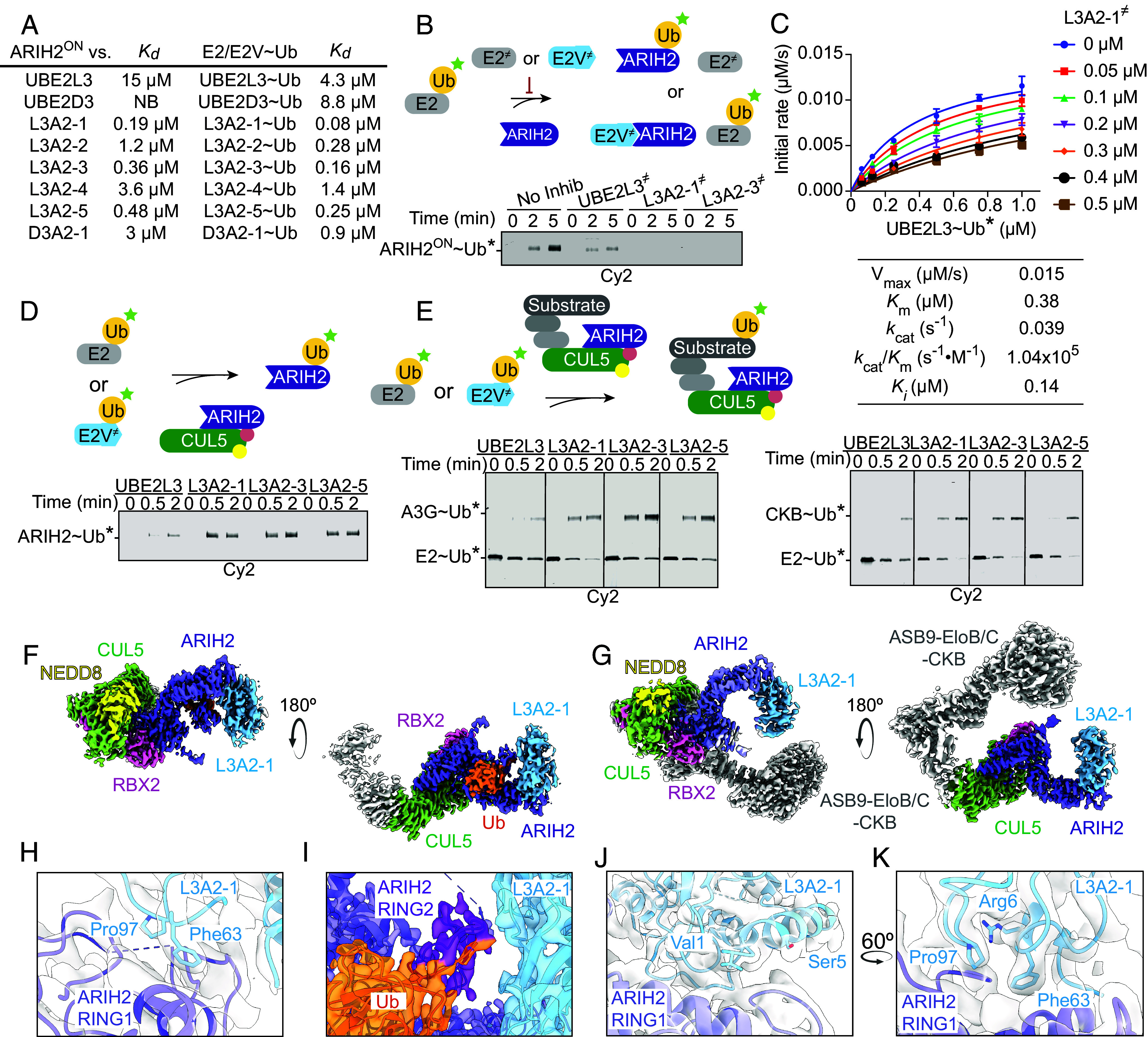
ARIH2^ON^ E2V reveals interaction between Ub conjugating enzyme and ARIH2. (*A*) Dissociation constants (K_d_), measured by ITC, between ARIH2^ON^ and UBE2L3, UBE2D3, indicated E2Vs, and stable mimics of E2/E2V~Ub intermediates with isopeptide linkage between Ub C-terminus and a Lys replacement for the E2 or E2V active site Cys. (*B*) Fluorescent Ub (Ub*) transfer from UBE2L3 or E2Vs to ARIH2^ON^. Ub* is detected on nonreducing SDS-PAGE (n = 2 technically independent experiments). (*C*) Kinetic parameters quantifying inhibition of fluorescent Ub (Ub*) transfer from UBE2L3 to ARIH2^ON^ by catalytically inactive L3A2-1 (L3A2-1^≠^, harboring an active site Cys to Ala substitution). Error bars represent the SD of two biological replicates for each reaction performed at indicated L3A2-1^≠^ concentrations (n = 2 technically independent experiments). (*D*) Nonreducing SDS-PAGE gels showing fluorescent Ub (Ub*) transfer from UBE2L3 or E2Vs to WT ARIH2 activated by neddylated CUL5-RBX2 (n = 2 technically independent experiments). (*E*) Neddylated CRL5/ARIH2-dependent fluorescent Ub (Ub*) transfer from UBE2L3 or indicated E2Vs to the substrates APBEC3G (recruited to CBFβ-HIV1 Vif) or CKB (recruited to ASB9) (n = 2 technically independent experiments). (*F*) Focused-refined cryo-EM map (from DeepEMhancer) representing Ub transfer from E2V L3A2-1 to ARIH2, through use of ABP that ultimately produces a crosslink between a modified C-terminus of Ub, the catalytic Cys86 of E2V L3A2-1, and the catalytic Cys310 of ARIH2 recruited to neddylated CRL5, ASB9, and its substrate CKB. The resolved portion of the map shows L3A2-1 in blue, Ub in orange, ARIH2 in purple, NEDD8 in yellow, CUL5 in green, and RBX2 in pink. (*G*) Cryo-EM map (from DeepEMhancer) showing E2V L3A2-1 binding to ARIH2 recruited to neddylated CRL5, ASB9, and its substrate CKB. The map is colored as in *F*, and with Elongin B, Elongin C, ASB9, and CKB homodimer in gray. (*H*) Close-up view of map in *F*, centered around the E2V L3A2-1 (blue) and ARIH2 RING1 (purple) interaction interface. As in canonical E2–RBR complexes, Pro97 and Phe63 contact with RING1 of ARIH2. (*I*) Close-up view of map in *F*, centered around the ubiquitin transfer catalytic center. (*J*) Close-up structure in *F*, focusing on the N-terminal helix region of L3A2-1 showing residues differing from WT UBE2L3 (Val1 and Ser5) facing ARIH2 RING1. (*K*) Same as *J*, with view rotated by approximately 60°, showing L3A2-1 Arg6 of N-terminal helix.

The E2Vs also showed 5- to 10-fold higher affinity for ARIH2^ON^ relative to their WT counterparts. The improved affinity was also observed for stable mimics of E2~Ub complexes (Fig. 4*A*). Catalytically inactive mutant versions (denoted with ≠) of the two highest-affinity E2Vs were also effective competitors of WT E2 activity, in that they were superior to mutant UBE2L3 in blocking UBE2L3-mediated ARIH2^ON^ autoubiquitylation (Fig. 4*B*). Quantifying this inhibition for the tightest-binding catalytically inactive E2V (L3A2-1^≠^) in steady-state kinetic assays revealed a K_i_ of 140 nM, which is similar to the 190 nM K_d_ for L3A2-1 measured by ITC (Fig. 4*C*).

Meanwhile, testing the Ub conjugating activity of three highest-affinity binders (L3A2-1, L3A2-3, L3A2-5) showed them to be more active than WT UBE2L3 in mediating ARIH2 autoubiquitylation (Fig. 4*D*). These E2Vs were also superior to WT UBE2L3 at catalyzing ARIH2 and neddylated CUL5-dependent ubiquitylation of substrates (CKB and APOBEC3G) recruited to the ASB9-Elongin B/C and VIF/CBFβ CRL substrate receptors, respectively (Fig. 4*E*).

We considered that the improved affinity of an E2V could enable obtaining a structure of ARIH2 bound to a Ub carrying enzyme, which has not to date been achieved with WT E2s by us or, to our knowledge, by others. After screening several complexes, we obtained cryo-EM structures of complexes containing a neddylated CUL5-RBX2 CRL (with substrate receptor ASB9 and its substrate CKB), ARIH2, and the E2V L3A2-1 (76, 77). A complex with only the E2V was refined at 3.06 Å resolution, and another chemically stable mimic of the ubiquitylation intermediate where Ub is covalently linked to both the E2V’s and ARIH2’s catalytic cysteine was refined at 2.97 Å resolution (Fig. 4 *F* and *G* and *SI Appendix*, Figs. S5 and S6 and Table S1) (78–80). Superimposing the ARIH2-E2V cryo-EM maps with prior structures by alignment over their E2-binding RING1 domains leads to three major conclusions. First, in agreement with much mutational analysis, an E2~Ub intermediate (here the E2V) binds ARIH2’s RING1 domain similarly to other RBR RING1 domains. Specifically, the E2V Phe63 and Pro97 insert between the ARIH2 RING1 hydrophobic cleft, essentially sealed there by the adjacent E2/E2V N-terminal helix (Fig. 4*H*). Second, the ARIH2-neddylated CUL5-RBX2 structure is similar before and during binding to an E2V, with the exception that the RING2 domain is uniquely visible when captured at the E2~Ub active site (Fig. 4*I*). The E2V~Ub-RBR conformation superimposes well with prior structures of activated RBR E3–E2~Ub complexes (*SI Appendix*, Fig. S4*B*). Third, the E2V-RING1 orientations subtly differ between the two complexes. Notably, such variations are also seen upon comparing different structures of UBE2L3-PARKIN, and of UBE2L3–ARIH1 complexes, and highlight the potential for dynamic RBR E3–E2 interactions (*SI Appendix*, Fig. S4*C*) (5, 7, 16–18).

Interestingly, although the two E2V residues differing from UBE2L3 (M1V and R5S) are located adjacent to the ARIH2 RING1 domain, the structures neither showed these residues making obvious interactions, nor showed them making contacts that would not be attainable by the WT sequence (Fig. 4*J*). We considered the possibility that the increased affinity and specificity of the E2V could inadvertently arise in the context of the specific N-terminal remnant sequence appended due to production as fusion proteins, but confirmed that the enhanced properties of the E2V were retained in the context of various N termini (*SI Appendix*, Fig. S4 *D*–*F*). Interestingly, prior structures have shown Arg5 directly interacting with RING1 domains of several RBR E3s, including HOIL1, HOIP, and PARKIN, but such interactions were not observed for complex representing Ub transfer from WT UBE2L3 to ARIH1(5, 7, 16–18, 42, 69). Thus, one explanation for the enhanced specificity, based on both the structural data and the sequences of E2Vs arising from our screens, could be that ARIH-family RBR E3s (and ANKIB1) can better tolerate substitution for Arg5. Notably, the adjacent residue, Arg6, is well defined at the center of the interface, between the E2V loops containing Phe63 and Pro97 and the ARIH2 RING1 domain (Fig. 4*K*). We speculate that subtle alterations in the conformational preferences of the E2V versus WT UBE2L3 (71, 81) could arise in superior positioning of Arg6, Phe63, Pro97, and adjacent residues to preferentially stabilize the interaction with ARIH2^ON^. It is also possible that differences in solvation could account for the increased affinity, or that the native E2 Met1 and Arg5 would contact ARIH2 in a way that reduces other optimal interactions such that their mutation results in increased affinity.

### E2V Derived ABPs Enable Active RBR E3 Ligase Profiling in Cells.

**Fig. 5.**
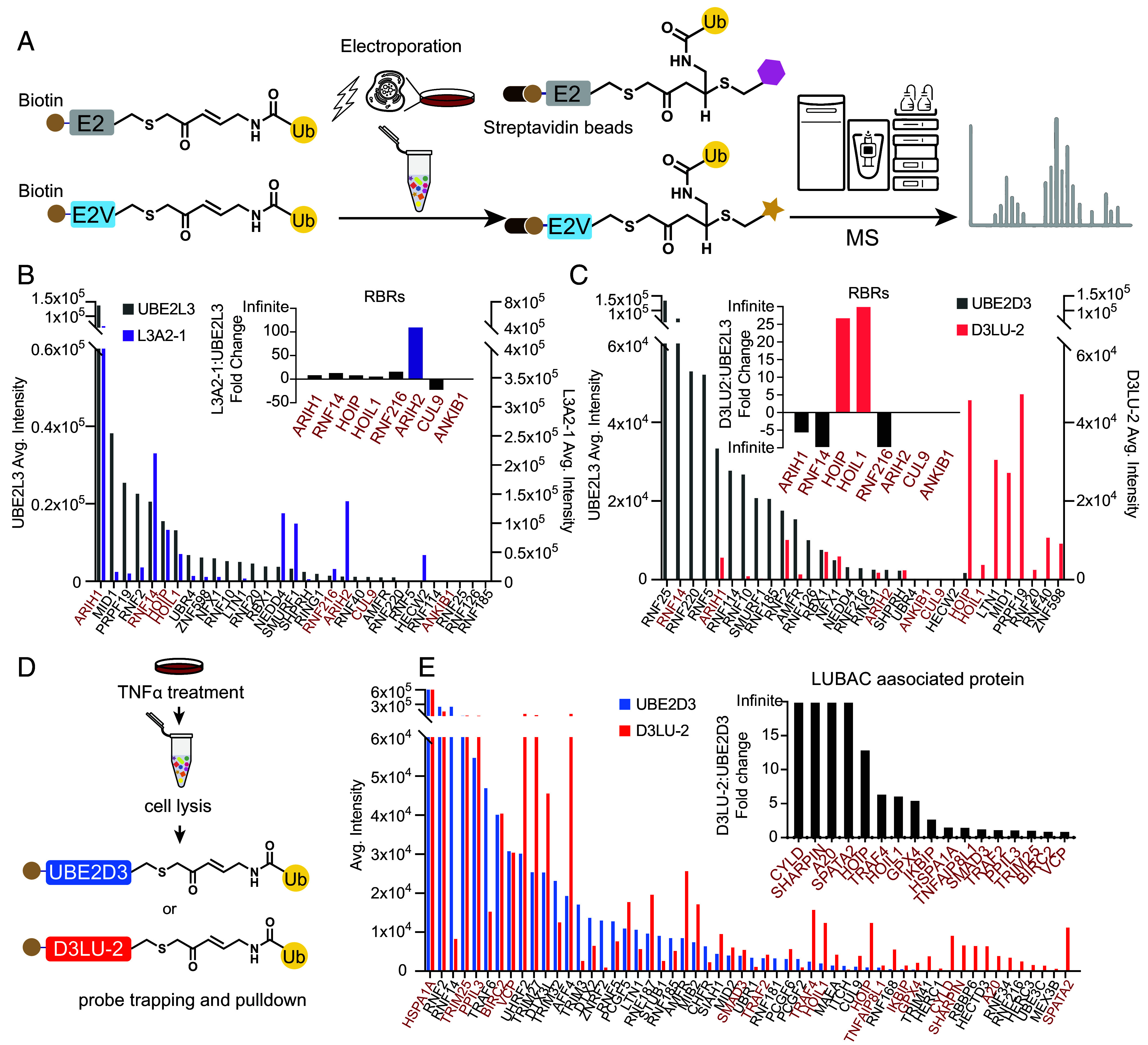
Profiling of activated RBR E3 ligases using E2V derived ABP. (*A*) Scheme for use of E2 and E2V derived ABPs for cellular RBR E3 ligase “profiling.” Biotinylated E2 or E2V are employed in ABP with electrophilic warhead between their catalytic Cys and Ub’s C-terminus. ABPs are introduced into cells by electroporation for covalent capture of E3s in the cell. After cell lysis, ABPs and their associated proteins are affinity enriched by magnetic streptavidin beads, followed by proteomic analysis by mass spectrometry. (*B*) E3 ligases captured by ABPs with UBE2L3 (gray) or the ARIH2^ON^-selected E2V L3A2-1 (purple). The X-axis shows E3s enriched by either ABP, with RBR E3s indicated in red. The Y-axis shows the average intensity. The *Inset* shows the fold change of RBR E3s detected with ABPs with E2 or E2V. Positive value indicates enrichment by E2V-containing ABP, and negative value indicates enrichment by wild-type E2-containing ABP. Bar graphs show the mean value of four biological replicates (n = 4) for each affinity-enriched mass spectrometry measurement. (*C*) Experiment as in *B*, except comparing ABPs employing WT UBE2D3 (gray) versus LUBAC E2V D3LU-2 (salmon). Infinite value indicates the protein is identified in either E2V (positive infinite) or wild-type E2 (negative infinite). (*D*) Scheme of E3 ligase profiling experiment with WT UBE2D3- or D3LU-2-derived ABPs after treatment of HEK293T cells with TNFα. (*E*) Proteins captured by ABPs with UBE2D3 ABP (blue) or the LUBAC-selected E2V D3LU-2 (red). The X-axis shows proteins enriched with either ABP, with known LUBAC components or associated proteins labeled in red. The Y-axis shows the average intensity of identified proteins. The *Inset* shows the fold change of known LUBAC-associated proteins captured by ABPs with either wild-type E2 or the LUBAC-selected E2V. Positive value indicates enrichment by E2V ABP, and negative value indicates enrichment by wild-type E2 ABP. Positive infinite value indicates the protein is only identified in experiments with E2V. Bar graphs show the mean value of four biological replicates (n = 4) for each pulldown mass spectrometry measurement.

We sought to gauge whether the sequence differences in E2Vs translate into altered interactions at a broader scale. Thus, we selected the highest affinity E2Vs for ARIH2^ON^ (L3A2-1) and LUBAC (D3LU-2) to create biotinylated ABPs for delivery into HEK293T cells by electroporation, and use as handles for affinity purification-mass spectrometry to identify interacting E3s (Fig. 5*A*) (82). This approach allowed identification of ~30 E3 ligases using probes generated with WT E2s (UBE2L3 or UBE2D3). The probes generated with the E2Vs each captured ~40 E3 ligases, and with different relative preferences. Overall, use of probes with different E2s and E2Vs expands the capacity to capture E3s. Furthermore, use of the probe containing the L3A2-1 E2V enabled 100-fold greater enrichment of ARIH2 compared to that with WT UBE2L3 (Fig. 5*B*). Similarly, HOIP and HOIL1 were selectively enriched by the probe containing the LUBAC-selected E2V D3LU-2, although other E3s were enriched as well (Fig. 5*C*). In addition to HOIL1 and HOIP, D3LU-2 enriches RING-type E3s, including LTN1, MID1, and PRPF19. Sequence alignment shows minimal similarity between the RING domains of these E3 ligases and RING1 domains of HOIL1 and HOIP (*SI Appendix*, Fig. S7*A*). However, comparing AlphaFold3-predicted RING domains of MID1 and LTN1 with the RING1 domain of HOIL1 and HOIP showed overall structural similarity. The loops in RING domains of MID1 and LTN1 adopt conformations similar to the RING1 loop of HOIP, particularly in the region that makes contact with Region 3 of UBE2D2, where D3LU-2 mutations are located (pink, *SI Appendix*, Fig. S7*B*). This structural similarity may account for the enrichment of these RING-type E3 ligases by the D3LU-2 probe.

Since LUBAC plays an important role in TNFα signaling, we tested whether our LUBAC E2V derived ABPs could enrich LUBAC upon pathway activation. HEK293T cells were treated with TNFα for 10 min prior to cell lysis (Fig. 5*D* and *SI Appendix*, Fig. S7*C*). We performed E3 ligase profiling using the probes generated with biotinylated WT E2s, or the E2Vs selected with LUBAC. Our data showed that TNFα treatment altered both the total proteome and the spectrum of proteins enriched by the ABPs generated with the WT UBE2D3 and with an E2V (*SI Appendix*, Fig. S7 *D*–*F*). Interestingly, there were, on average, around 6,000 proteins identified in the total proteome with and without TNFα treatment, but neither HOIL1 nor HOIL were detected among these, suggesting their relatively low abundance in the cellular environment, and value in using ABPs to enrich them for profiling. Most strikingly, compared with the ABP with WT UBE2D3, that with the D3LU-2 E2V selectively enriched LUBAC E3s and several proteins previously reported to bind LUBAC, including CYLD, A20, and SPATA2 (83) (Fig. 5*E*). Thus, the use of E2V-based ABPs have distinctive capacity to assess activation of specific signaling pathways.

## Discussion

In summary, by varying native E2 sequences, and performing a series of positive and negative selections, we developed a suite of probes with increased affinity and specificity for their targeted RBR E3 ligases (Fig. 1*A* and *SI Appendix*, Fig. S1*F*). These tools offer a range of enhanced functionalities, including superior ubiquitylation activity, or in some cases in the context of active site mutants, superior E3 ligase inhibition in vitro. The distinct specificities also allowed generation of E2s with substantially enhanced selectivity for each of the two RBR E3 ligases within the LUBAC, while the improved affinity allowed obtaining a cryo-EM structure where such attempts with the WT E2s have to date not yielded success.

One unexpected finding was that we did not generally obtain E2Vs that were entirely specific for their targeted RBR E3 ligase, despite performing extensive negative selection. We note that our assays for specificity are extremely stringent, as they are performed with pure protein at higher protein concentrations than standard ELISA screens. In many cases, cross-reactivity was greatest across RBR subfamilies, such as the ARI-family (6). Notably, we previously observed the same trend for UbV cross-reactivity toward HECT E3s within a subfamily (60), although it is also possible that our libraries simply did not access sequences that would have achieved absolute specificity.

It was also surprising that the residues altered in the structurally characterized E2V do not directly contact the targeted E3 (ARIH2) (Fig. 4*J*). The results highlight the complexity of protein–protein interactions. Interactions may be enhanced by means other than addition of direct contacts, for example, by removal of residues that are suboptimal for highest affinity docking of key residues. It seems likely that our discovery of such an E2V depended on our experimental selection protocol. It will be interesting to see in the future if computational approaches to designing proteins with altered selectivity capture such nuanced contributions to affinity and specificity.

Another unexpected finding was that, for many of the E2Vs, the increased affinity correlated with increased ubiquitylation flux, not only from the E2V active site to the E3 but also when tested in the context of multiturnover assays examining the transfers of several ubiquitins to substrates (of CUL9) (Fig. 2 *C* and *D*) or linear polyubiquitylation by HOIP or LUBAC. Thus, it is possible to select for E2Vs that increase reactivity with targeted E3s while maintaining ability to be repeatedly charged by E1s, at least under conditions of some in vitro assays.

Finally, use of E2Vs in ABPs with a reactive Ub linked at catalytic Cys enables capture of distinct cohorts of the cellular collection of ubiquitylating enzymes. In this context, E2Vs have capacity to outperform their parental E2s in capturing their targeted RBR E3s, including those upregulated along with their partner proteins in response to a stimulus. Like the previously described E2~Ub probes (46, 84, 85), E2V probes capture a variety of HECT and RBR E3s and the E1 enzyme UBA1, presumably through their catalytic Cys, as well as other proteins that react with the Ub warhead. As no single E2 or E2V probe developed to date uniformly captures all such enzymes, it may be that broader coverage can be achieved by performing such reactive E3 profiling with pooled collections of probes in the future, including those harboring UBE2L3, UBE2D-family E2s, and those with E2Vs. We propose that our strategy for selecting E2Vs could be applied to other E3s in the RBR, HECT, and RCR E3 families. Moreover, our strategy could in principle be tweaked, through generation of new E2V libraries combining residues we found most frequently altered in the successful hits, and by varying the cohorts of E3s used in positive and negative selection. This could yield probes with varying selectivity for a large fraction of catalytic cellular E3s participating in thioester transfer reactions. Furthermore, use of phage display to select for an E2 with increased E1 specificity has also been reported (86). Due to the generalizable nature of our approach, which could in principle be applied to all E3s functioning in E1–E2–E3 cascades, we anticipate that the E2V technology will find application ranging from structural and mechanistic studies to mapping activation status of entire families of ubiquitin ligases in signaling cascades.

## Methods

### Construction of E2V Libraries and Phage Display Selection against RBR E3s.

Phage-displayed libraries were constructed as previously described (87, 88). The hard randomization was implemented using oligonucleotides containing hand-mixed degenerate codons to cover all 20 natural amino acids at every position of mutagenized residues (Integrated DNA Technologies). Libraries were constructed using site-directed mutagenesis targeting each region of the E2 binding hotspot (89). The screens were performed with a combination of three independently generated libraries based on each E2 scaffold, where the variable positions (*SI Appendix*, Fig. S1 *B* and *E*) in Region 1, 2, or 3 were designed to encode all possible residues. The libraries for UBE2L3 regions 1, 2, and 3 have theoretical diversities of 20^5^, 20^4^, and 20^7^, respectively. The libraries for UBE2D3 Regions 1, 2, and 3 have theoretical diversities of 20^5^, 20^4^, and 20^5^, respectively. The phage input used for each selection was 10^11^, in an effort to maximize coverage of the library. Oligonucleotide design and combinations are described in *SI Appendix*, Fig. S1*D* and Dataset S1. Phage display selection and phage ELISA procedures are provided in *SI Appendix*, *Supplemental Methods*.

### Cloning, Protein Expression, and Purification.

All RBR E3 ligases, Elongin B, Elongin C, ASB9, APOBEC3C, and CKB are of human origin. They were cloned into pGEX and pRSF vectors for expression in *Escherichia coli*; pFLN2 and pACEBac1 vector for expression in *Trichoplusia ni* High Five insect cells and pEG vector for expression in HEK 293S cells. All wild-type E2s, E2Vs, and NEDD8 were cloned into pGEX vector for expression in **E. coli*.* Untagged ubiquitin was cloned into pET3a vector for expression in *E. coli*. Detailed expression and purification procedures for each protein and biotinylation of E2 and E2Vs are provided in *SI Appendix*, *Supplemental Methods*.

### Ubiquitylation Assay and Inhibition Kinetics Analysis.

Ubiquitylation assay with ARIH1^ON^, ARIH2^ON^, ANKIB1, RNF14, and CUL9-RBX1 were performed in pulse–chase format and ubiquitylation assay with HOIL1, HOIP, and LUBAC were performed in multiturnover format. Inhibition kinetics with ARIH2^ON^ in presence of L3A2-1^≠^ was performed in pulse–chase format. Detailed experimental procedures are provided in *SI Appendix*, *Supplemental Methods*.

### In Vitro Pulldown of RBR E3s Using E2Vs.

C-terminally biotinylated E2Vs and their mutants (1 µg) were incubated with 7 µL of magnetic streptavidin beads (blocked with 1% BSA) in 20 µL of pulldown buffer (25 mM HEPES pH7.5, 150 mM NaCl, 2 mM DTT) for 30 min at 4 °C. The streptavidin beads were then washed 4 times with 200 µL pulldown buffer. Then, 1 µg of RBR E3 in 200 µL of pulldown buffer was incubated with the beads at 4 °C for 30 min. The beads were washed 5 times with 500 µL pulldown buffer, and captured E3s were eluted with 5× Laemmli loading buffer, separated on 4 to 20% SDS-PAGE, and visualized by Coomassie staining.

### Additional Methods.

Detailed experimental procedures for binding analysis of RBR E3, E2s, and E2Vs by ITC and BLI, cryo-EM sample preparation, data processing and model refinement, generation of ABPs, cell culture, and mass spectrometry measurement and analysis are provided in *SI Appendix*, *Supplemental Methods*.

## Supplementary Material

Appendix 01 (PDF)

Dataset S01 (PDF)

Dataset S02 (XLSX)

Dataset S03 (PDF)

## Data Availability

The atomic coordinates and cryo-EM maps have been deposited in the RCSB with accession codes PDB ID 9SDX (RBR binding E2 variant crosslinked with NEDD8-CUL5-RBX2 bound ARIH2 and Ub) (76), PDB ID 9SDY (RBR E2 variant binding to CUL5-RBX2 bound ARIH2) (77) and EMDB with codes EMD-54793 (RBR binding E2 variant crosslinked with NEDD8-CUL5-RBX2 bound ARIH2 and Ub) (78), EMD-54794 (RBR E2 variant binding to CUL5-RBX2 bound ARIH2) (79), EMD-54795 (Focused ASB9-EloB/C-CKB bound to NEDD8-CUL5-RBX2-ARIH2-E2 variants) (80). The proteomics data have been deposited in PRIDE with accession number PXD068594 (82). E2 variants plasmids are available from Addgene with accession number 248581 for L3A2-1 (90), 248586 for L3A2-3 (91), 248587 for L3A1-1 (92), 248589 for L3R14-1 (93), 248590 for L3HP-1 (94), 248591 for D3LU-2 (95), 248846 for L3A2-2 (96), 248847 for L3A2-4 (97), 248848 for L3A2-5 (98), 248849 for D3A2-1 (99), 248850 for L3R14-2 (100), 248851 for L3C9-1 (101), 248852 for L3AN-1 (102), 248853 for L3AN-2 (103), 248854 for L3A1-2 (104), 248855 for L3HL-1 (105), 248856 for L3LU-1 (106), 248857 for L3LU-2 (107), 248858 for D3LU-1 (108). Sequencing data for these plasmids are also available on their respective Addgene pages. Uncropped raw gels with molecular weight markers and Coomassie Blue stained Fluorescent scanned gels are provided in Dataset S1. Oligonucleotide design for phage library construction, ELISA, kinetics and annotated proteomics raw data are provided in Dataset S2. ITC and BLI data are provided in Dataset S3. All other data are included in the manuscript and/or supporting information.
