# Supplementary material for: E2 variants for probing E3 ubiquitin ligase activities
Source: Proc Natl Acad Sci U S A. 2026 Jan 2;123(1):e2524899122. doi: 10.1073/pnas.2524899122 (PMC12773759; doi:10.1073/pnas.2524899122)
Supplement: Supplementary file 1 — Appendix 01 (PDF) [file pnas.2524899122.sapp.pdf]

PNAS

Supporting Information for
E2 Variants for Probing E3 Ubiquitin Ligase Activities

Jiale Du[1], Gisele A. Andree[1], Daniel Horn-Ghetko[1], Luca Stier[1,2], Jaspal Singh[3], Sebastian Kostrhon[1], Leo Kiss[1], Matthias Mann[4], Sachdev S. Sidhu[3]* and Brenda A. Schulman[1,2]*

1 Department of Molecular Machines and Signaling, Max Planck Institute of Biochemistry, Martinsried, Germany, 82152

2 Department of Chemistry, School of Natural Sciences, Technical University of Munich, Garching, Germany, 85748

3 School of Pharmacy, University of Waterloo, Waterloo, Ontario, Canada, N2L 3G1

4 Department of Proteomics and Signal Transduction, Max Planck Institute of Biochemistry, Martinsried, Germany, 82152

*Corresponding Authors: schulman@biochem.mpg.de and sachdev.sidhu@uwaterloo.ca

This PDF file includes:

Supplemental Methods
Figures S1 to S7
Table S1
Legends for Datasets S1 to S3
SI References

Other supporting materials for this manuscript include the following:

Datasets S1 to S3

Supporting Information Text

Supplemental Methods

Phage enzyme-linked immunosorbent assays (ELISA) to assess scaffold display

Phage ELISA was performed as previously described (1). Briefly, 96-well plates were coated with anti-M13 antibody at 10 µg/mL in PBS overnight at 4 ºC and blocked with blocking buffer (0.5% BSA in PBS) for 2 h at room temperature. Plates were washed 4 times with PT buffer (PBS, 0.05% Tween 20). Phage supernatants 2-fold serially diluted in PBT buffer (PBS, 0.5% BSA, 0.05% Tween 20) were added to plates and incubated for 1h. After washing 4 times with PT buffer, anti-FLAG-HRP (Thermo Scientific) secondary antibody was added and plates were incubated for 30 minutes. Plates were washed 4 times with PT buffer, and TMB substrate (Thermo Scientific) was added. After 10 min incubation for color development, the reaction was stopped by addition of phosphoric acid and absorbance was measured at 450 nm. Individual steps were performed at room temperature unless otherwise indicated.

Phage display selections against RBR E3s

Phage-displayed UBE2L3 and UBE2D3-based E2V libraries were used for selections against a panel of activated RBR E3s: ARIH1^{ON}, ARIH2^{ON}, ANKIB1, HOIL1, HOIP, LUBAC, RNF14, CUL9-RBX1, and RNF216. E3 proteins were individually immobilized on 96-well Maxisorp plates (Invitrogen) by incubating with 0.5 µM of E3 for 30 min at 4 ºC with agitation. For the first two rounds of selection, negative selection was performed prior to positive selection. For negative selection, the pooled E2V libraries consisting of one of the three RBR binding regions mutated were sequentially incubated with plates immobilized with each non-target RBR E3 for 30 min at 4 ºC, and the flowthrough was collected for the subsequent negative selection. For LUBAC, the non-target RBRs are only HOIL1 and HOIP, instead of the entire panel of RBR E3s, to ensure selection of E2V binding to LUBAC as a complex instead of HOIL1 and HOIP on their own. Following the final non-target RBR negative selection step, the phage library flowthrough was transferred to the plate immobilized with the target RBR E3 and incubated for 1 h at 25 ºC. Unbound phages were removed by washing 10 times with PT buffer (PBS, 0.02% Tween-20). Bound phages were eluted and amplified overnight for the next round of selection. After five rounds of selection (2 negative-positive, and 3 positives only), 40 individual colonies were inoculated for analysis by ELISA, and positive clones were sequenced. Convergent sequences were cloned into bacterial expression vectors, and only clones that expressed in *E. coli* were picked for further biochemical analysis.

Expression and purification of RBR E3s and substrate binding modules

RBR E3s were expressed in *E. coli*, *Trichoplusia ni* High Five insect cells, and HEK 293S cells. The activated variants of ARIH1 and ARIH2 were referred to as "ON", which are activated by mutations that relieve autoinhibition (F430A/E431A/E503A in ARIH1, L381A/E382A/E455A in ARIH2). ARIH1^{ON} carrying N-terminal GST tag with a subsequent TEV cleavage site, wild type ARIH2 and ARIH2^{ON} carrying N-terminal His-MBP tag with a subsequent TEV cleavage site, HOIL1 carrying N-terminal 8xHis tag with a subsequent TEV cleavage site, catalytic domain of HOIP (696-1072) carrying N-terminal 8xHis-SUMO tag with a subsequent SENP2 cleavage site, ASB9 carrying N-terminal GST tag with a subsequent TEV cleavage site, untagged EloB/C and CKB carrying N-terminal GST tag with a subsequent TEV cleavage site were expressed in E. coli cells, and APOBEC 3C carrying N-terminal GST tag with a subsequent TEV cleavage site was expressed in *Trichoplusia ni* High Five insect cells as previously described (2, 3). ANKIB1 lacking the C-terminal disordered region (1-778) carrying N-terminal GST tag with a subsequent TEV cleavage site, RNF14 carrying N-terminal TwinStrep tag with a subsequent 3C protease cleavage site and RNF216 carrying N-terminal TwinStrep tag with a subsequent 3C protease cleavage site were all expressed in *Trichoplusia ni* High Five insect cells. Untagged HOIP, HOIL1 carrying N-terminal 8xHis tag with a subsequent TEV cleavage site and SHARPIN carrying N-terminal TwinStrep tag with a subsequent 3C protease cleavage site was cloned into pACEBac1 vectors for co-expression in insect cell. RBX2 carrying N-terminal GST tag with a subsequent TEV

cleavage site and untagged CUL5 were also co-expressed in insect cell as previously described (2). RBX1 carrying N-terminal GST tag with a subsequent TEV cleavage site and untagged CUL9 were co-expressed in mammalian cells (HEK293S) to yield a complex that is activated by neddylation of CUL9 as previously described (4). His-lipoyl domain-tagged TP53 was expressed and purified as previously described (4).

Protein expressing cells were harvested by centrifugation and resuspended in lysis buffer (50 mM HEPES pH 7.5, 200 mM NaCl, 1 mM DTT, 1x cOmplete™ Protease Inhibitor, Roche), followed by sonication and centrifugation for 30 min at 50,000 xg. For proteins carrying an N-terminal TwinStrep-tag the supernatant was incubated with Strep-Tactin Sepharose resin (Cube Biotech) for 1 h at 4°C, followed by washing with 20 bead volumes of wash buffer (50 mM HEPES pH 7.5, 200 mM NaCl, 1 mM DTT) and elution with 2.5 mM desthiobiotin in wash buffer. For proteins carrying an N-terminal GST-tag, the supernatant was incubated with Glutathione Sepharose 4B resin (Cytiva) for 1 h at 4°C, followed by washing with 20 bead volumes of wash buffer. Proteins were eluted with 10 mM glutathione in wash buffer. For proteins carrying an N-terminal 8xHis-tag, 8xHis-MBP tag, and 8xHis-SUMO tag, the supernatant was incubated with PureCube 100 Ni-INDIGO Agarose (Cube Biotech) for 1 h at 4°C, followed by washing with 20 bead volumes of wash buffer containing 20 mM imidazole. Proteins were eluted with 300 mM imidazole in wash buffer. The tags were cleaved by incubating either the beads or the eluted proteins with the respective proteases overnight at 4°C (PreScission 3C, TEV proteases at 1:50 protein:protease molar ratio, SENP2 protease at 1:25 protein:protease molar ratio). The proteins purified through 8xHis-tag were purified by anion exchange chromatography on HiTrap QP HP column (Cytiva) with a linear gradient 2-55% Buffer QB (25 mM HEPES pH 7.5, 1M NaCl, 1mM DTT) with Buffer QA (25 mM HEPES pH 7.5, 1mM DTT) prior to size exclusion chromatography. Final purification step for all the proteins was size-exclusion chromatography on a Superose 75 Increase 10/300 GL column (Cytiva) using SEC buffer (25 mM HEPES pH 7.5, 150 mM NaCl, 1 mM DTT).

For LUBAC, tandem affinity purification was performed using Ni-INDIGO Agarose pulldown for His tagged HOIL1, followed by Strep-Tactin pulldown for SHARPIN. Affinity purified LUBAC was then purified by anion exchange chromatography on HiTrap QP HP column and size-exclusion chromatography on a Superose 200 Increase 10/300 GL column (Cytiva).

Expression and purification of E2Vs

E2V hits were cloned into the bacterial expression plasmid pGEX carrying an N-terminal GST-tag and a subsequent TEV cleavage site. *E. coli* BL21 (DE3) cells harboring the expression plasmid were grown in LB supplemented with ampicillin at 100 µg/mL overnight at 37 ºC, 200 rpm. The starter culture was diluted 1:100 into Terrific Broth/Amp and cultured at 37 ºC, 200 rpm until the OD reached 0.6. Protein expression was induced with IPTG to a final concentration of 400 µM, and cultures were grown overnight at 18 ºC, 200 rpm. Cell pellets were collected by centrifugation at 5,000 xg for 15 min, resuspended in lysis buffer (50 mM HEPES pH 7.5, 150 mM NaCl, 1 mM DTT, 1 mM PMSF), and sonicated for 6 min at 30% amplitude using a 3-second pulse cycle. The lysates were centrifuged at 50,000 xg for 30 minutes and the supernatants were incubated with Glutathione Sepharose resin (Cytiva) for 1h at 4ºC. The resin was then washed with 20 bead volumes of binding buffer (25 mM HEPES pH7.5, 150 mM NaCl, 1 mM DTT), followed by overnight on-bead digestion with 2 µM TEV protease at 4 ºC. The eluates were concentrated and further purified by size exclusion chromatography on a Superose 75 Increase 10/300 GL column (Cytiva) equilibrated in SEC buffer. The peak fractions were collected and concentrated using Amicon® Ultra Centrifugal Filters (Merck Millipore) with a 10 kDa molecular weight cut-off.

Biotinylation of E2Vs

The Avi-tag was cloned at the C-terminus of E2Vs. Biotinylation reactions were carried out by mixing 5 mg/mL Avi-tagged E2Vs, 5 mM $MgCl_2$, 2 mM ATP, 150 µM biotin, and 2 µM BirA in PBS and incubating with shaking at 30 ºC for 1 h. The reactions were then purified by size exclusion chromatography on a Superose 75 Increase 10/300 GL column (Cytiva) equilibrated in SEC buffer. The peak fractions were collected and concentrated using Amicon® Ultra Centrifugal

Filters (Merck Millipore) with a 10 kDa molecular weight cut-off. Biotinylation of E2Vs was confirmed by intact mass spectrometry.

Expression and purification of ubiquitination and neddylation machinery and fluorescent labeling of Ub

Untagged WT ubiquitin was expressed in *E. coli* Rosetta (DE3) cells and purified as previously described (5). In short, 0.6% perchloric acid was slowly added to the cell lysate until a pH of ~4.5 was reached. This precipitated most other proteins but ubiquitin. The supernatant was dialyzed into 25 mM sodium acetate pH 4.5, 100 mM NaCl, cleared by centrifugation and further purified by ion exchange chromatography on a HiTrap SP HP column (Cytiva), followed by size exclusion chromatography on a Superdex75 10/300 GL column (Cytiva) into 25 mM HEPES pH 7.5, 150 mM NaCl, 1 mM DTT.

N-terminal His tagged UBA1, UBA3, N-terminal GST tagged wild type UBE2L3, UBE2D3, UBE2E1, UBE2L3 mutants (catalytic Cys86 only, catalytic C86A, F63A), UBE2D3 mutants (catalytic Cys85 only, catalytic C85A, F62A), NEDD8, UBE2F and Ub were all expressed and purified as previously described.(2, 3) Ub with an additional N-terminal Cys is used for fluorescent labeling to get Fluorescent Ub (Ub*) as previously described (2–4).

E2~Ub conjugating assay

E2 charging assay with UBE2L3 and RNF14 E2Vs (L3R14-1 and L3R14-2) were performed in presence or absence of catalytically inactive RNF14 (RNF14C417A, labeled as RNF14$^{\neq}$). Briefly, reactions were prepared by mixing 2 μM E2 or E2V and 15 μM fluorescently labeled Ub, with or without 2 μM RNF14$^{\neq}$ in reaction buffer (25 mM HEPES pH 7.5, 100 mM NaCl, 2.5 mM $MgCl_2$, 2.5 mM ATP). Reactions were started by addition of 0.1 μM UBA1, incubated at RT, and quenched at indicated time points with non-reducing 6x SDS-PAGE sample buffer to be able to visualize formation of E2~Ub thioester linked intermediates.

Multi-turnover ubiquitylation assay

All ubiquitylation reactions with the HOIL1, HOIP and LUBAC were performed in a multi-turnover format. Reactions were prepared by mixing 1 μM E2 or E2V, 1 μM HOIP, or LUBAC, and 15 μM labeled or unlabeled Ub in reaction buffer (25 mM HEPES pH 7.5, 100 mM NaCl, 2.5 mM $MgCl_2$, 2.5 mM ATP). Reactions were started by addition of 0.2 μM UBA1, incubated at RT, and quenched at indicated time points with 6x SDS-PAGE sample buffer. Products were separated by SDS-PAGE gel and imaged with an Amersham Typhoon imager (using the Cy2 channel) to visualize turnover of fluorescently-labeled ubiquitin. The sugar ubiquitination assays with HOIL1 and LUBAC included additionally 20 mM maltoheptaose (Sigma), and unlabeled linear tri-Ub or N-terminal fluorescently labeled linear tri-Ub instead of Ub. Formation of ubiquitinated maltoheptaose was visualized by either Coomassie staining or Cy2 fluorescent scanning.

Pulse-chase ubiquitylation assay

All ubiquitylation reactions with ARIH1^{ON}, ARIH2^{ON}, ANKIB1, RNF14 and CUL9-RBX1 were performed in pulse-chase format as previously described (2–4) with the following modifications. Pulse mixes were prepared in 10 μL reaction volume by mixing 0.3 μM UBA1, 2 μM E2 or E2V, and 15 μM fluorescent labeled or unlabeled Ub in pulse buffer (25 mM HEPES pH 7.5, 100 mM NaCl, 2.5 mM $MgCl_2$, 2.5 mM ATP) for 30 min at RT. The reaction was then quenched by addition of 15 μL Quench buffer (25 mM HEPES pH 7.5, 100 mM NaCl, 83 mM EDTA). The pulse mix was then incubated on ice for 5 min. A 3 μL aliquot of pulse mix were then diluted into 15 μL of chase reaction mix (25 mM HEPES pH 7.5, 100 mM NaCl, 50 mM EDTA), and a zero-time point was taken by mixing 3 μL of reaction with 7 μL of SDS-PAGE sample buffer. The chase reaction was initiated by addition of 0.5 μM E3 diluted in chase buffer (25 mM HEPES pH 7.5, 100 mM NaCl). The reaction was incubated at RT and quenched at indicated time points with non-

reducing 6x SDS-PAGE sample buffer to be able to visualize also E2~Ub thioester linked intermediates.

Kinetic analysis of ARIH2 ubiquitin transfer inhibition by catalytically inactive E2V

Inhibition kinetic analysis was performed in pulse chase assay format as described above. Various concentrations of UBE2L3~Ub* (0.125 µM, 0.25 µM, 0.5 µM, 1 µM, 1.5 µM and 2 µM) were generated by incubating UBE2L3 with 0.3 µM E1 and 10 µM fluorescent Ub* for 30 min at 25 ºC, followed by addition of quench mix and dilution into chase reaction mix. Various amount of catalytically inactive L3A2-1 were added to chase mix containing 0.2 µM ARIH2^{ON} to reach final concentration of 0.1 µM, 0.2 µM, 0.4 µM, 0.6 µM and 0.8 µM and 1 µM L3A2-1$^{\neq}$. The chase reactions were initiated by adding 10 µL chase mix into 10 µL chase reaction mix. Reaction was quenched after 7 s with 3x non-reducing SDS-loading buffer. Along with ARIH2~Ub* standards (0.1 µM, 0.05 µM, and 0.025 µM), generated by reacting 1 µM E2~Ub* with limited ARIH2 (0.1 µM, 0.05 µM, and 0.025 µM) for 10 min at 25 ºC, reaction products were separated by 15% SDS-PAGE gel. Gels were imaged using a Typhoon FLA 9500 (GE Healthcare) on Cy2 channel to visualize turnover of fluorescently-labeled ubiquitin. Images were analyzed using ImageJ to quantify the intensity of ARIH2~Ub*. The initial rate of ARIH2~Ub* formation was calculated in concentration per second (µM/s). These values were fitted to the Michealis-Menten equation and competitive inhibition model to obtain K_m and k_{cat} and K_i value in GraphPad Prism10. Data are presented as mean ± standard deviation of three replicates. Kinetics raw data are provided in Dataset S2.

Isothermal titration calorimetry (ITC) analysis

ITC measurements were performed on a MicroCal PEAQ-ITC (Malvern) at 25 °C with a setting of 19 × 2 µL injections. RBR E3s, E2s and E2Vs were all dialyzed into dialysis buffer (25 mM HEPES pH 7.5, 150 mM NaCl, and 0.5 mM TCEP) prior to analysis. For measurements, the syringe contained E2 or E2Vs at concentrations of 300-500 µM, and the cell contained RBR E3 proteins at 10-25 µM. The heats of dilution from diluting E2s into the measurement buffer were subtracted from the binding experiments before curve fitting. Manufacturer-supplied software was used to fit the data to a single-site binding model and to determine the stoichiometry (N), ΔH, ΔS, and the association constant K_a. The dissociation constant, K_d, was calculated from K_a. ITC data are provided in Dataset S3.

Biolayer interferometry (BLI) analysis

BLI measurements were performed on an 8-channel Octet® R8 system (Sartorius) at 25°C. First, Octet® Streptavidin Biosensors (Sartorius) were hydrated in the equilibration buffer (25 mM HEPES pH 7.5, 150 mM NaCl, 2mM DTT, 0.05% Tween20) for 30 min at 25 ºC, and then loaded with fixed concentration of N-terminal biotinylated ARIH2^{ON} (200 µL, 2.5 µg/mL). Various concentrations of E2 and E2Vs were 1:2 serial diluted 8 times from 50 µM. Measurements were initiated with baseline correction in the equilibration buffer, followed by association phase by dipping biotin-ARIH2^{ON} loaded biosensor into wells containing E2 and E2Vs. Then the biosensors were dipped again into the equilibration buffer to measure dissociation phase. K_d was calculated by measuring the k_{on} and k_{off} during the association and dissociation phase. BLI data are provided in Dataset S3.

Sample preparation and cryo-EM data collection for NEDD8-CUL5-ARIH2-E2Vs

For L3A2-1-Ub covalently trapped samples, 2 µM neddylated CUL5-RBX2, 2 µM ARIH2 were mixed in trap reaction buffer (25 mM HEPES pH 8.0, 100 mM NaCl), and 10 µM L3A2-1~Ub trap was added. The reaction was incubated at 25 ºC for 15 min. Then, 4 µM ASB9-EloB/C, and 4 µM CKB were added to the reaction and incubated on ice for 10 min, followed by size exclusion chromatography on a Superose 6 10/300 GL column (Cytiva). Peak fractions containing NEDD8-CUL5-RBX2-ARIH2~L3A2-1~Ub-ASB9-EloB/C-CKB were pooled and concentrated to 3.5 mg/mL (Fig. S6A-C). For noncovalent complex, purified 2 µM neddylated CUL5-RBX2, 2 µM ARIH2, 10 µM L3A2-1, 4 µM ASB9-EloB/C, 4 µM CKB were incubated on ice for 20 min followed by size

exclusion chromatography on a Superose 6 10/300 GL column (Cytiva). Peak fractions containing NEDD8-CUL5-RBX2-ARIH2-L3A2-1-ASB9-EloB/C-CKB were pooled and concentrated to 4 mg/mL (Fig. S7A-B). Shortly before plunging, n-Octyl-β-D-glucopyranoside (β-OG) was added to the protein sample to a final concentration of 0.05%. This was essential to overcome preferred orientation of the sample. Subsequently, holey carbon grids (Quantifoil, R1.2/1.3, 200 mesh) were glow discharged, and 3.5 µL of sample was applied to the grid at 95% humidity and 4°C using a Vitrobot Mark IV (Thermo Fisher Scientific) and plunge-frozen into liquid ethane (blot force 3, blot time 3 s). High-resolution data were collected on a Titan Krios transmission electron microscope (TEM) equipped with a post-GIF Gatan K3 Summit direct electron detector operating in counting mode. Movies were collected at a nominal magnification of 105,000x, equaling to 0.8512 Å/pixel at the specimen level. The target defocus ranged between -1.0 and -2.6 µm, and the total dose of 60 $e^-/Å^2$ was distributed over 40 frames.

Processing cryo-EM maps of ARIH2-L3A2-1 complexes

Data processing was performed using cryoSPARC version 4.2 (6) and workflow are shown in Fig. S6D-G and S7C-F. Briefly, raw movies were imported into cryoSPARC, and patch motion correction and patch contrast transfer function (CTF) estimation were performed. Automated blob particle picking was performed with 180-220 Å diameter, followed by micrograph curation. Curated micrographs were used for all subsequent processing steps, including template picking, two-dimensional classification, heterogenous refinement, three-dimensional classification, non-uniform refinement, global CTF refinement, local refinement, and final post-processing with DeepEMhancer. The statistics of refined cryo-EM maps are provided in Table S1.

Model building and refinement

Model building of the NEDD8-CUL5-RBX2-ARIH2-L3A2-1-ASB9-EloB/C-CKB and NEDD8-CUL5-RBX2-ARIH2~L3A2-1~Ub structures were based on published structures of NEDD8-CUL5-RBX2-ARIH2 (PDB: 7ONI), ASB9-EloB/C and CKB (PDB: 6V9I, 6V9H), and ARIH1-UBE2L3 (PDB: 7B5N). Briefly, individual chains from the published NEDD8-CUL5-RBX2-ARIH2 or ASB9-EloB/C-CKB structures were docked into the refined cryo-EM maps. The E2V and Ub densities were initially docked by aligning the ARIH2 RING1 with the ARIH1 RING1, and then UBE2L3 and Ub were fitted into the densities corresponding to L3A2-1 and Ub, respectively. The composite models were then split into several cohesive domains or segments and docked into the cryo-EM maps using ChimeraX (7). The models were refined through manual building in Coot (version 0.8.9.2) (8) alternating with iterations of real-space refinements with Phenix refinement (9). For regions where residues could not be unambiguously assigned, the published structures were aligned to nearby well-resolved residues to help in building the backbone of the poorly resolved region, and the side chains of these residues were removed. The chemical ligand used to cross-link L3A2-1, ubiquitin and ARIH2 (identified in the PDB file by the compound 'SY8') was first modelled in the correct orientation and proximity with L3A2-1's active site Cys86. The position of a single Cys residue of L3A2-1 corresponding to ARIH2's active site Cys310 and Ub C-terminal Gly75 to complete the three-way cross-link was estimated based on the position of the ligand relative to ARIH1's active site Cys and UBE2L3's active site Cys in the ARIH1-UBE2L3-Ub structure (PDB: 7B5N). Then the bond angle between the ligand and ARIH2, Ub and L3A2-1 were then refined using Real Space refinement on Phenix with bond angle restrains applied. The statistics of final models are provided in Table S1.

Generation of activity-based probes (ABPs)

Ub-MESNa was first generated as previously described (3). Briefly, N-terminal 6xHis tagged Ub lacking its C-terminal glycine 76 (Ub^{1-75}) intein-chitin binding domain (CBD) fusion protein was expressed in E. coli pRIL cells at 30 ºC for 4 h after induction with 300 µM IPTG, and lysed in 25 mM MES pH 6.2, 100 mM NaCl, and 2.5 mM PMSF. The cell lysate was clarified and bound to chitin resin for 1 h at 4ºC. Next, 100 mM MESNa was added to cleave CBD fused to Ub^{1-75} intein and release Ub1-75-MESNa. After overnight incubation at room temperature, Ub^{1-75}-MESNa was purified by size exclusion chromatography on a HiLoad Superdex 75 column. The purified Ub1-75

MESNa was then coupled to (E)-3-[2-(bromomethyl)-1,3-dioxolan-2-yl] prop-2-en-1-amine (BmDPA) (>95% purity, ChiroBlock) to yield reactive Ub-BmDPA as previously described (3, 10–13). Subsequently, the reactive Ub-BmDPA was mixed with 2-fold excess wild-type E2 or E2V containing a single catalytic cysteine with or without a C terminal Avi-tag, and incubated for 1 h at 30 °C. The reaction products were further purified by nickel affinity pulldown on His tagged Ub and size-exclusion chromatography, yielding E2-Ub or E2V-Ub ABPs.

Cell culture

HEK293T cells were cultured in Dulbecco's Modified Eagle Medium (DMEM, Gibco) containing 10% fetal bovine serum (Gibco), 4 mM GlutaMAX (Gibco), 1 mM sodium pyruvate, 100 units/mL penicillin and 100 μg mL−1 streptomycin (Gibco) at 37 °C with 5% CO2. Cells were grown in 10 cm dishes. For TNFα treatment, cells were first serum starved for 3 h, then incubated with 25 ng/mL TNFα at RT for 10 min. Subsequently, cells were washed and harvested in PBS (Gibco).

Electroporation for ABP cellular delivery and proteomics

The Neon® Transfection System (Thermo Fisher Scientific) was used to perform all electroporations. HEK293T cells were washed with PBS (Gibco) while adherent, then dissociated with TrypLE™ Express (Gibco) and washed again with PBS in suspension before resuspending in Buffer R (Thermo Fisher Scientific). 1 x 106 cells in a volume of 11 µL were added to 2 µL of 52 µM E2V. The sample was loaded into a 10 µL Neon® Pipette Tip, electroporated at 1400V for 20 ms with 2 pulses, then transferred to 0.5 mL of pre-warmed antibiotic-free medium. Samples were incubated with open lids at 37°C with 5 % CO2 for 15 min before washing once with PBS and exposure to an 'N8-block' treatment (14), 3-minute incubation with 1 µM MLN4924 (MedChemExpress, HY-70062) and 1 µM CSN5i-3 (MedChemExpress, HY-112134) in PBS, followed by an additional PBS wash, before snap-freezing the cell pellet in liquid nitrogen.

ABP pulldown-MS

Cells were lysed in lysis buffer containing 25 mM HEPES pH 7.5, 5% glycerol, 150 mM NaCl, 0.5% NP-40, 1x HALT protease/phosphatase inhibitor (Thermo Fisher Scientific), 2 µM MLN4924 (MedChemExpress, HY-70062), 2 µM CSN5i-3 (MedChemExpress, HY-112134). Lysates were cleared by spinning at 21,300xg at 4 °C for 3 minutes, and the supernatant was filtered with a 0.22 µm cellulose acetate Costar centrifuge tube filter (Corning) at 16,000xg for 2 minutes before transfer to a Protein LoBind tube (Eppendorf). High Capacity Magne® Streptavidin Beads (Promega) were washed twice in HBS (HEPES Buffered Saline), then once in HBS with 0.5% BSA, before resuspension in 25 mM HEPES pH 7.5, 5% glycerol, 150mM NaCl, 0.5% NP-40. Washed beads were added to the cleared lysates at a ratio of 0.3 µL bead volume per 1 µg electroporated E2V, and incubated for 30 minutes at 4 °C with rotation. After incubation, beads were washed twice with 25 mM HEPES pH 7.5, 5% glycerol, 150mM NaCl, 0.5% NP-40, then transferred to a new Protein LoBind tube. Next, beads were washed twice with 25 mM HEPES pH 7.5, 1% SDC, and transferred to a new Protein LoBind tube. Finally, beads were washed once with HBS, and once with distilled water, transferred to a new Protein LoBind tube, resuspended in 100mM Tris pH 8, 1% SDC, and boiled for 5 minutes at 95 °C. After cooling to room temperature, TCEP (VWR) and 2-Chloroacetamide (Sigma Aldrich) were added to final concentrations of 10 mM and 40 mM, respectively. The sample was incubated for 5 minutes at 95 °C with rotation at 1,000 rpm, then cooled to room temperature. Overnight on-bead digestion was performed at 37 °C with rotation at 1,000 rpm using 0.5 µg each of Trypsin and LysC per sample. On the following day, beads were removed by centrifugation before the addition of TFA to a final concentration of 1% in each sample. Samples were cleared by spinning at 20,000xg at 4 °C for 5 min, and the peptide concentration was determined using either BCA assay (Thermo Fisher Scientific) or tryptophan fluorescence assay (15).

LC-MS/MS measurements

Samples were run on an Evosep One LC unit (EvoSep, EV-1000) connected to a TimsTOF Pro 2 mass spectrometer (Bruker Daltonics) via a CaptiveSpray ion source fitted with a 10 µm fused

silica inner-diameter emitter (Bruker Daltonics, 1865691). Chromatographic separation was performed using a 30 samples per day program on a 15 cm x 150 µm column packed with 1.9 µm C18 beads (Bruker Daltonics, 1893471), and maintained at a constant temperature of 50°C. The mobile phase system comprised buffer A (0.1% formic acid in water) and buffer B (0.1% formic acid in acetonitrile). Data acquisition followed a dia-PASEF workflow involving 20 scans, each incorporating two ion-mobility windows, to cover the m/z range of 350–1,200. Isolation window widths were determined using the py_diaid tool (16). Ion mobility was set between 0.7 and 1.3 V s cm^{-2}, with both the ion accumulation and ramp phases fixed at 100 ms. Collision energy was applied as a linear gradient, starting from 20 eV at $1/K_0$ = 0.6 V s cm^{-2} and reaching 59 eV at $1/K_0$ = 1.6 V s cm^{-2}.

MS data analysis

Raw data were processed using DIA-NN 1.9.2 for precursor and fragment identification, searching against the reviewed human proteome (Uniprot, November 2024, 20,663 entries without isoforms) in library free mode (17). A maximum of two variable modifications were allowed and variable modifications for methionine oxidation, N-terminal acetylation, and cysteine carbamidomethylation were set. MBR and 'deep-learning-based spectra, RT, and IM prediction' were also enabled. Using Python (3.13.5) and the packages pandas (2.3.0) and directlfq (0.3.2), protein intensities were quantified using directLFQ and filtered for 100% valid values in at least one condition (18). Intensities for each identified protein group were further visualized in GraphPad Prism (10.5.0). For each identified protein, filters were applied based on the intensity of the control experiment using a UBE2L3 mutant which is unable to bind RING1 domain of RBR E3. Only proteins with lower than 5 % intensity in the negative control compared to wild-type E2 or E2Vs were identified as positive hits and plotted in bar graph using GraphPad Prism (10.5.0). Tabulated mass spectrometry data are provided in Dataset S2 and the raw proteomics data have been deposited in PRIDE with accession number PXD068594

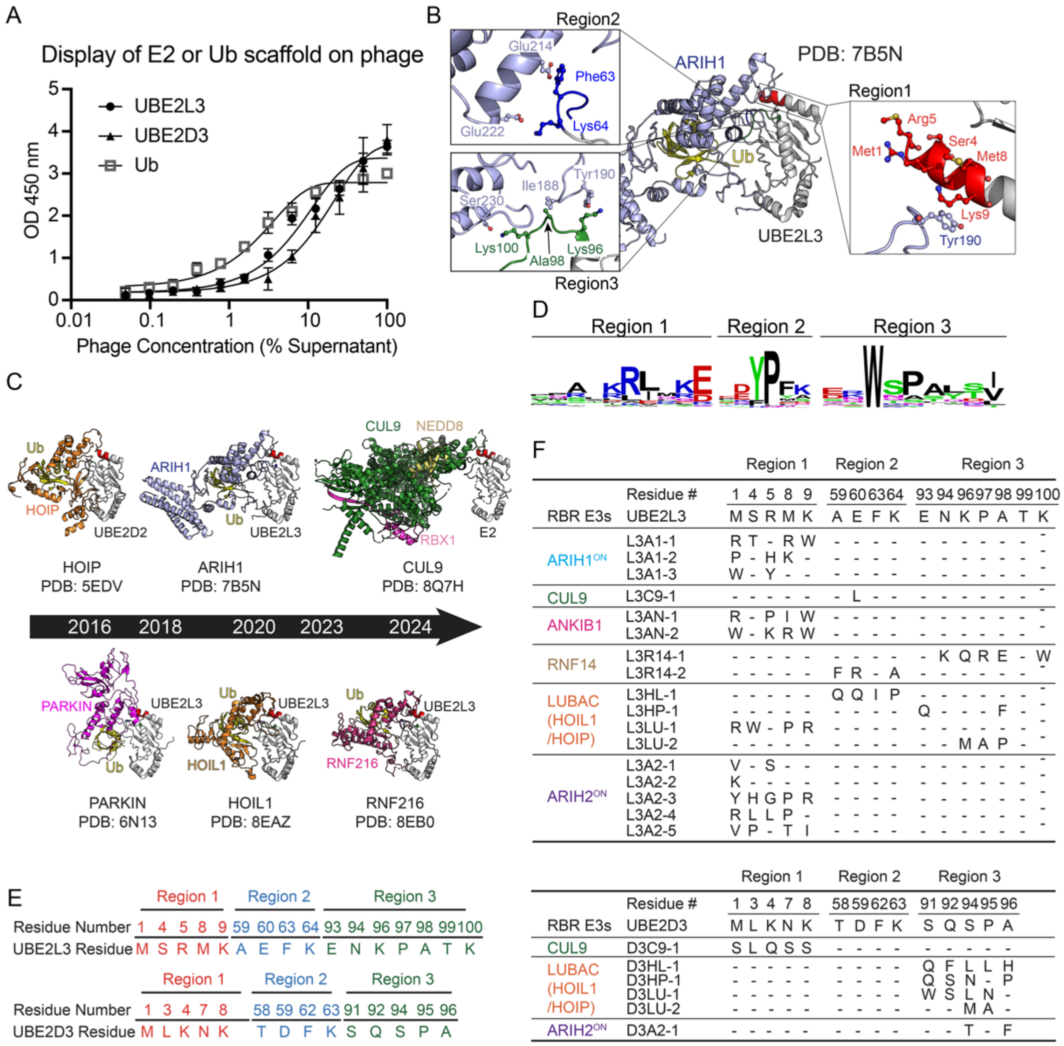

Fig. S1. Design of E2V library based on E2 scaffold

(A) Phage Enzyme-Linked Immunosorbent Assay (ELISA) showing expression of E2s and Ub scaffold on bacteriophage surface. Error bars represent the standard deviation (SD) of three biological replicates for absorbance measurement at 450nm at each phage dilution (n = 3 technically independent experiments).
(B) Structure of activated ARIH1 covalently crosslinked to UBE2L3~Ub showing RBR E3 and UBE2L3 binding interface (PDB: 7B5N). The RBR-interacting interface on UBE2L3 is clustered into three regions: Region 1 (red) is a helix, Region 2 (blue) and Region 3 (green) are loops.
(C) Chronological report of structures on RBR E3s in complex with Ub conjugating enzymes UBE2L3 or UBE2D2 (PDB:5EDV, 6N13, 7B5N, 8EAZ, 8EB0, 8Q7H).
(D) Sequence logo of three regions of human E2 enzymes.
(E) The residues of the three RBR binding regions of UBE2L3 and UBED3 picked for randomization to generate the E2V library.
(F) E2V hits for RBRs from libraries based on UBE2L3 (top) and UBE2D3 (bottom).

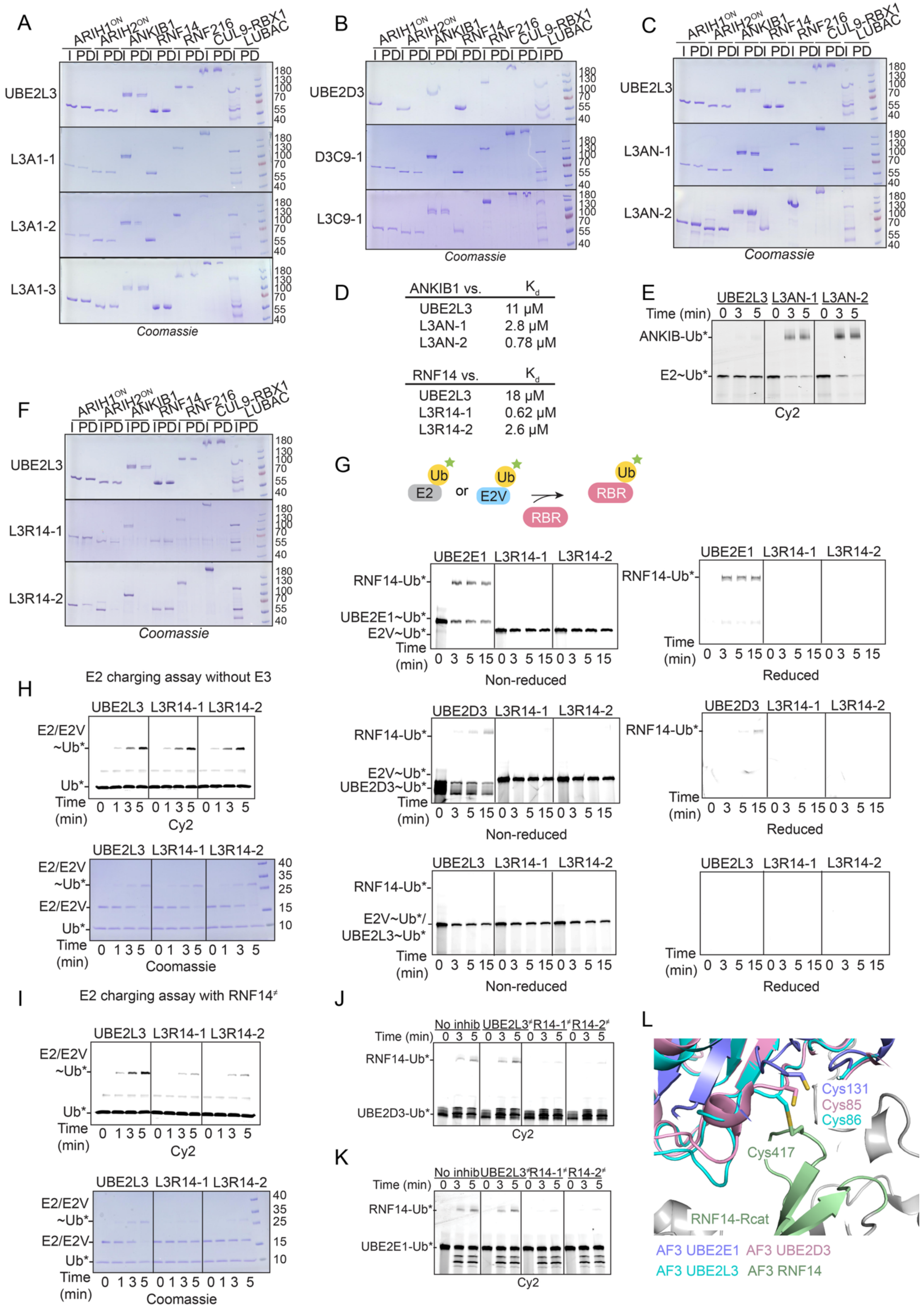

A
B
C
D
E
F
G
H
I
J
K
L
UBE2L3
L3A1-1
L3A1-2
L3A1-3
UBE2D3
D3C9-1
L3C9-1
L3AN-1
L3AN-2
L3R14-1
L3R14-2
Coomassie
ANKIB1 vs. Kd
UBE2L3 11 μM
L3AN-1 2.8 μM
L3AN-2 0.78 μM
RNF14 vs. Kd
UBE2L3 18 μM
L3R14-1 0.62 μM
L3R14-2 2.6 μM
ANKIB-Ub*
E2~Ub*
Cy2
RNF14-Ub*
UBE2E1~Ub*
E2V~Ub*
Non-reduced
Reduced
E2 charging assay without E3
E2 charging assay with RNF14♯
E2/E2V ~Ub*
Ub*
Time (min)
No inhib
UBE2D3-Ub*
UBE2E1-Ub*
Cys131
Cys85
Cys86
Cys417
RNF14-Rcat
AF3 UBE2E1
AF3 UBE2D3
AF3 UBE2L3
AF3 RNF14

Fig. S2. E2Vs for ANKIB1 and RNF14 slightly improve the selectivity and ubiquitylation activity

(A) Coomassie-stained gels showing indicated RBR E3s in input (I) or after affinity pulldown (PD) by E2 or E2Vs selected for binding ARIH1^{ON}. LUBAC consists three proteins, HOIL1, HOIP and SHARPIN are all visible in gel portion shown here (n = 2 technically independent experiments).
(B) Experiment as in **A**, except with E2Vs selected for binding CUL9-RBX1.
(C) Experiment as in **A**, except with E2Vs selected for binding ANKIB1.
(D) Dissociation constants (K_d) between ANKIB1 and UBE2L3 or ANKIB1 E2Vs (top) and between RNF14 and UBE2L3 or RNF14 E2Vs (bottom) measured by isothermal titration calorimetry (ITC).
(E) Fluorescent Ub (Ub*) transfer from UBE2L3 or E2Vs to ANKIB1. Ub* is detected on non-reducing SDS-PAGE (n = 2 technically independent experiments).
(F) Experiment as in **A**, except with E2Vs selected for binding RNF14.
(G) Fluorescent Ub (Ub*) transfer from UBE2E1, UBE2D3, UBE2L3 or RNF14 E2Vs to RNF14. Ub* is detected on non-reducing or reducing SDS-PAGE (n = 2 technically independent experiments).
(H-I) Fluorescent Ub (Ub*) transfer from E1 to UBE2L3 or RNF14 E2Vs in absence (H) or presence (I) of catalytic inactive RNF14 (RNF14$^{\neq}$). Ub* is detected on non-reducing SDS-PAGE (n = 2 technically independent experiments).
(J-K) Fluorescent Ub (Ub*) transfer from UBE2D3 (J) or UBE2E1 (K) to RNF14 in presence of either catalytically inactive UBE2L3 or E2Vs selected for binding RNF14. Ub* is detected on non-reducing SDS-PAGE (n = 2 technically independent experiments).
(L) AlphaFold-3 predicted structures of RNF14 (Gray), UBE2E1 (Purple), UBE2D3 (Pink) and UBE2L3 (Cyan) showing E2 active sites' interaction with RNF14 Rcat domain (Green).

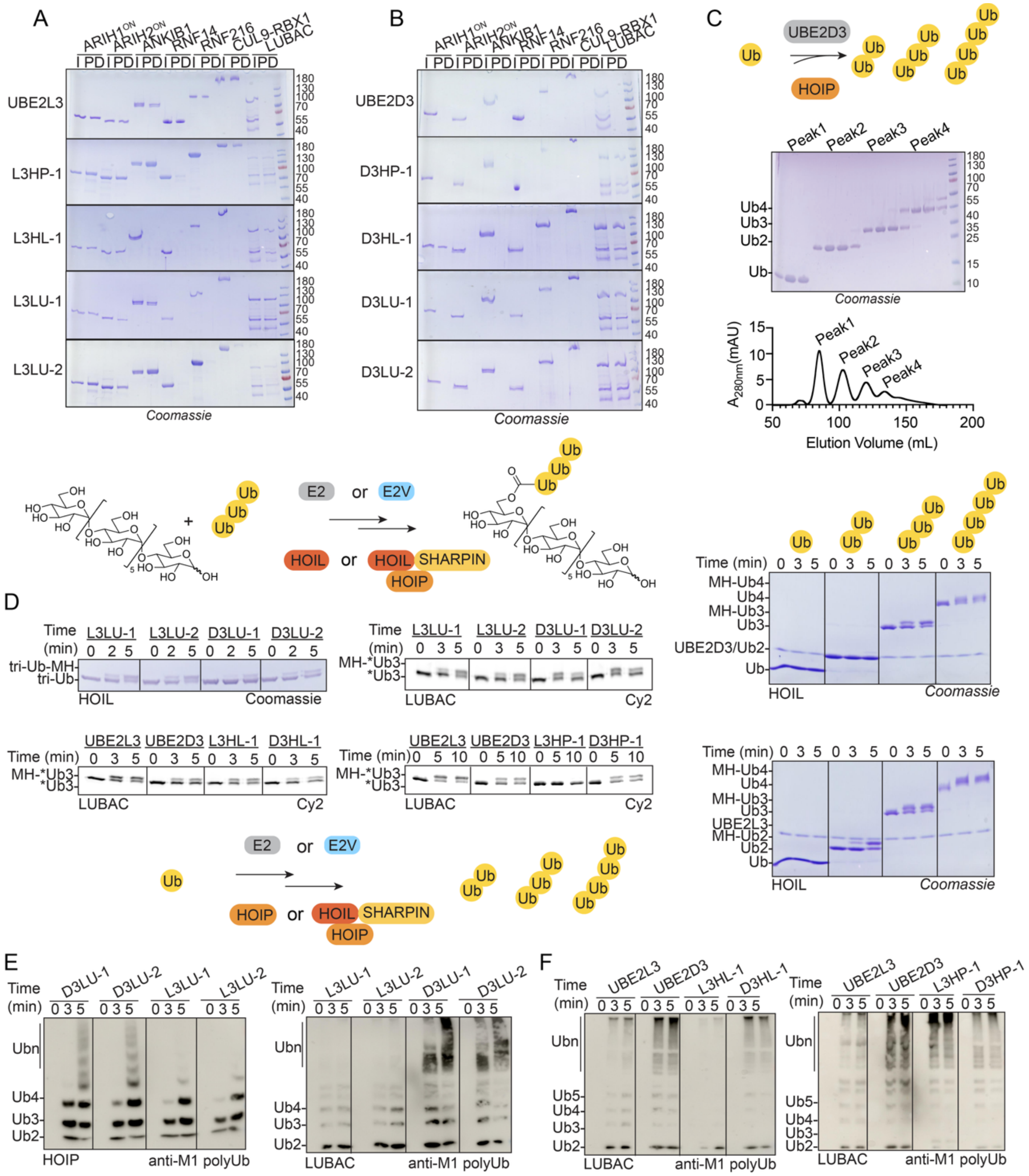

Fig. S3. E2Vs for HOIL1, HOIP and LUBAC process E3 selectivity.

(A) Coomassie-stained gels showing indicated RBR E3s in input (I) or after affinity pulldown (PD) by E2 or E2V selected for binding HOIP, HOIL or LUBAC (n = 2 technically independent experiments).
(B) Experiment as in **A**, except with E2Vs derived from UBE2D3.
(C) Linear Ub chains are formed after incubating 10 µM Ub with 1 µM UBE2D3 and 1 µM HOIP at 30 ºC for 15 min. Linear Ub chains are separated by cation exchange chromatography. Maltoheptaose (MH) ubiquitylation by HOIL1 or HOIP with WT UBE2L3 or UBE2D3. Reactions were performed with Ub or pre-assembled linear di-Ub, tri-Ub or tetra-Ub as the source of donor Ub (n = 2 technically independent experiments).
(D) Maltoheptaose (MH) ubiquitylation by HOIL1 or LUBAC with indicated E2Vs selected for binding HOIL1, HOIP or LUBAC. Reactions were performed with pre-assembled linear tri-Ub or

tri-Ub with distal Ub fluorescently labeled at N-terminus to block chain extension as the source of donor Ub (n = 2 technically independent experiments).
(E) Ubiquitin chain formation by HOIP or LUBAC with indicated E2Vs selected for binding LUBAC (n = 2 technically independent experiments).
(F) Experiment as in **E**, except with E2Vs selected for binding HOIL1 or HOIP (n = 2 technically independent experiments)

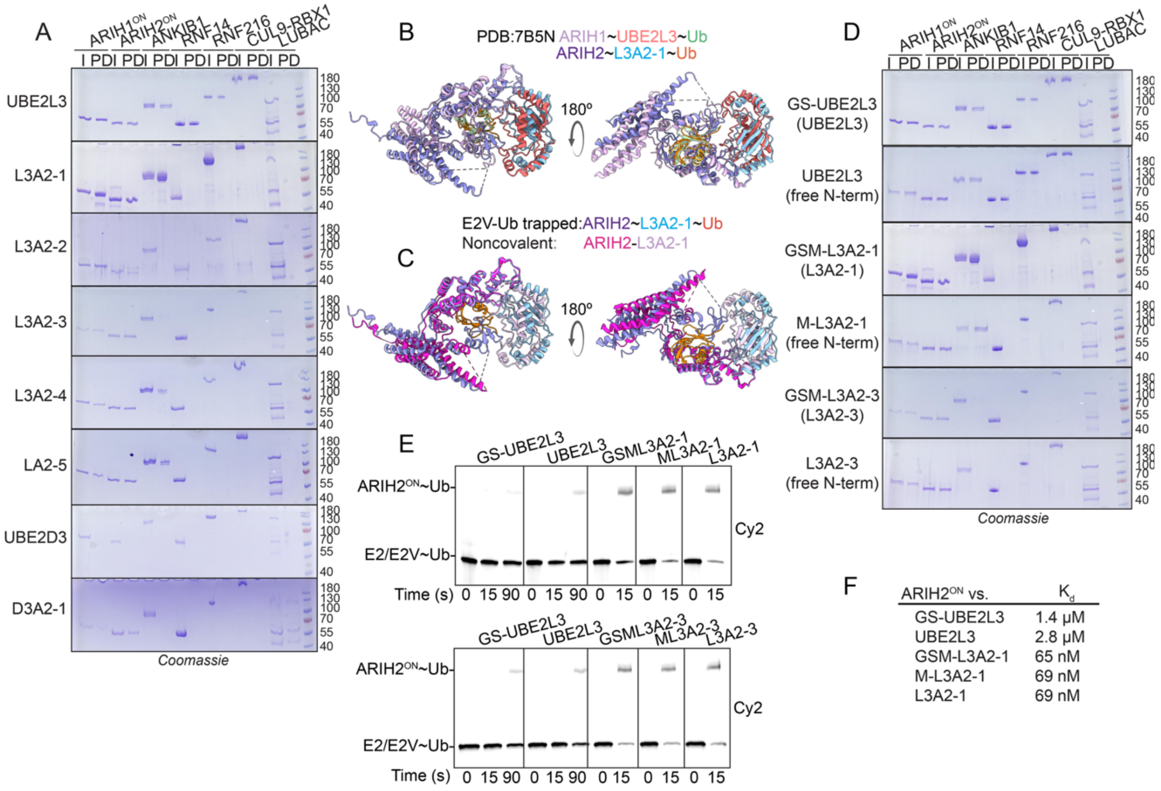

Fig. S4. ARIH2^{ON} E2Vs exhibit selectivity towards ARIH1 and ARIH2 and N-terminal insertions have minimal effect on E2 or E2V and ARIH2 interaction

(A) Coomassie-stained gels showing indicated RBR E3s in input (I) or after affinity pulldown (PD) by E2 or E2V selected for binding ARIH2^{ON} (n = 2 technically independent experiments).
(B) E2V maintenance of canonical structural mechanism, shown by superimposition of the structure of structure representing Ub transfer from E2V L3A2-1 to neddylated CRL5-activated ARIH2 with that representing Ub transfer from WT UBE2L3 to neddylated CRL1-activated ARIH1 (PDB: 7B5N). Both structures were captured by reaction with activity-based probes, and aligned over the RBR E3 RING1 domains. ARIH1 is shown in light pink, UBE2L3 in red, and Ub between them ARIH1 is in light green. ARIH2 is shown in purple, E2V L3A2-1 in blue, and Ub between them ARIH2 is in orange.
(C) Variable positioning of Ub conjugating enzyme and RBR E3, shown by superimposition of the structures in F and G aligned over ARIH2 RING1 domains.
(D) Experiment as in A, except with E2 or E2V with either N-terminus additional GS from TEV cleavage (-GS) or free N-terminus.
(E) Fluorescent Ub (Ub*) transfer from UBE2L3 or E2Vs with different N-terminus to ARIH2^{ON}. Ub* is detected on non-reducing SDS-PAGE (n = 2 technically independent experiments).
(F) Dissociation constants (K_d) between ARIH2^{ON} and UBE2L3 or E2Vs with different N-terminus measured by biolayer interferometry (BLI).

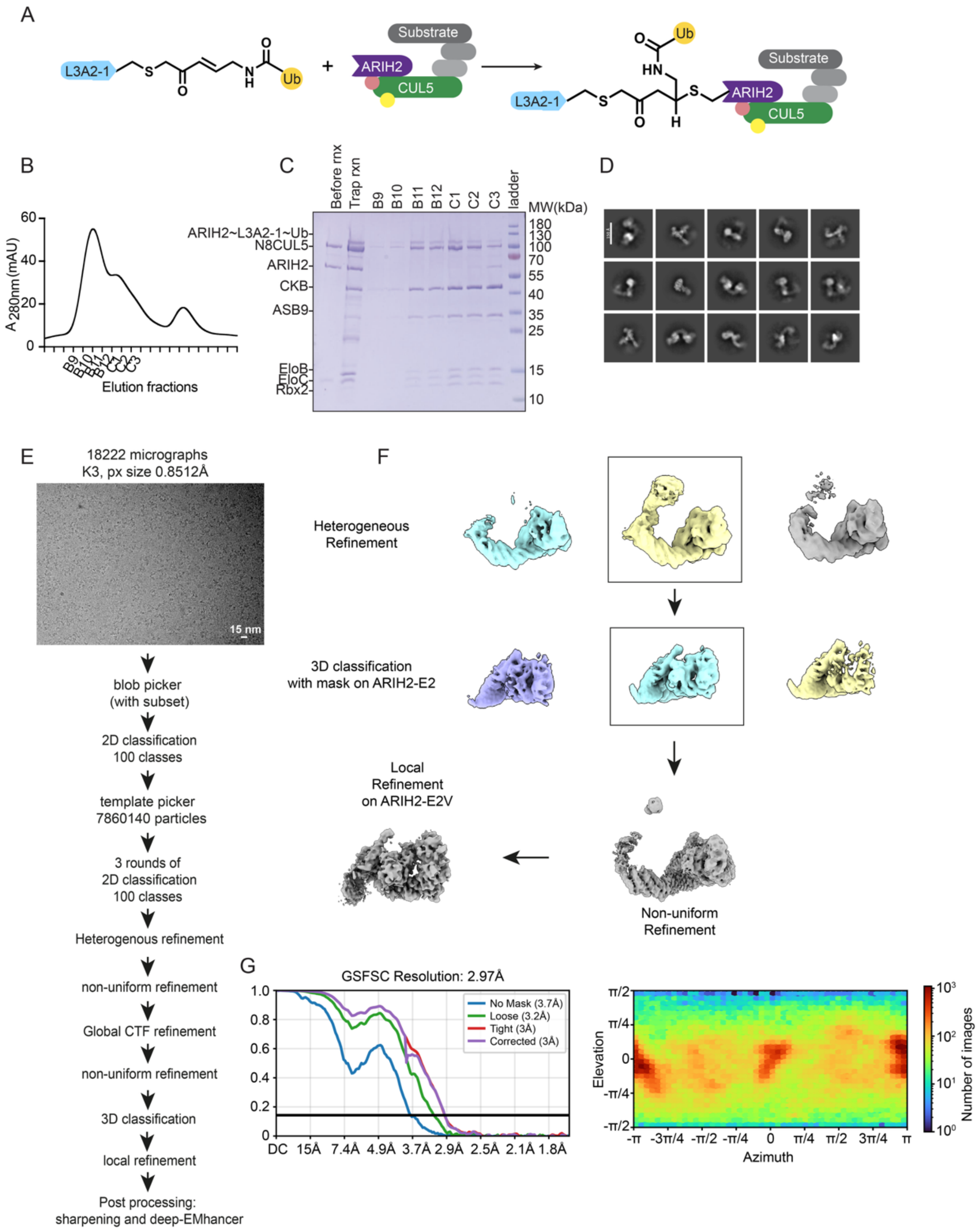

Fig. S5. Structural determination of ABS9-EloB/C-CKB-NEDD8-CUL5-RBX2-ARIH2~L3A2-1~Ub by cryo-EM

(A) Scheme of generation of ARIH2~L3A2-1~Ub intermediate by crosslinking ABS9-EloB/C-CKB-NEDD8-CUL5-RBX2 bound ARIH2 with L3A2-1~Ub.
(B-C) Purification of ABS9-EloB/C-CKB-NEDD8-CUL5-RBX2-ARIH2~L3A2-1~Ub (B) size exclusion chromatogram (C) SDS-PAGE analysis of input and fractions of size exclusion chromatography for cryo-EM.
(D) 2D classes of ABS9-EloB/C-CKB-NEDD8-CUL5-RBX2-ARIH2~L3A2-1~Ub complex.
(E) Cryo-EM processing workflow for ABS9-EloB/C-CKB-NEDD8-CUL5-RBX2-ARIH2~L3A2-1~Ub complex.

(F) Representative maps of heterogenous refinement, 3D classification and local refinement of ABS9-EloB/C-CKB-NEDD8-CUL5-RBX2-ARIH2~L3A2-1~Ub.
(G) GSFSC resolution estimation and angular distribution graph of final global refinement of ABS9-EloB/C-CKB-NEDD8-CUL5-RBX2-ARIH2~L3A2-1~Ub.

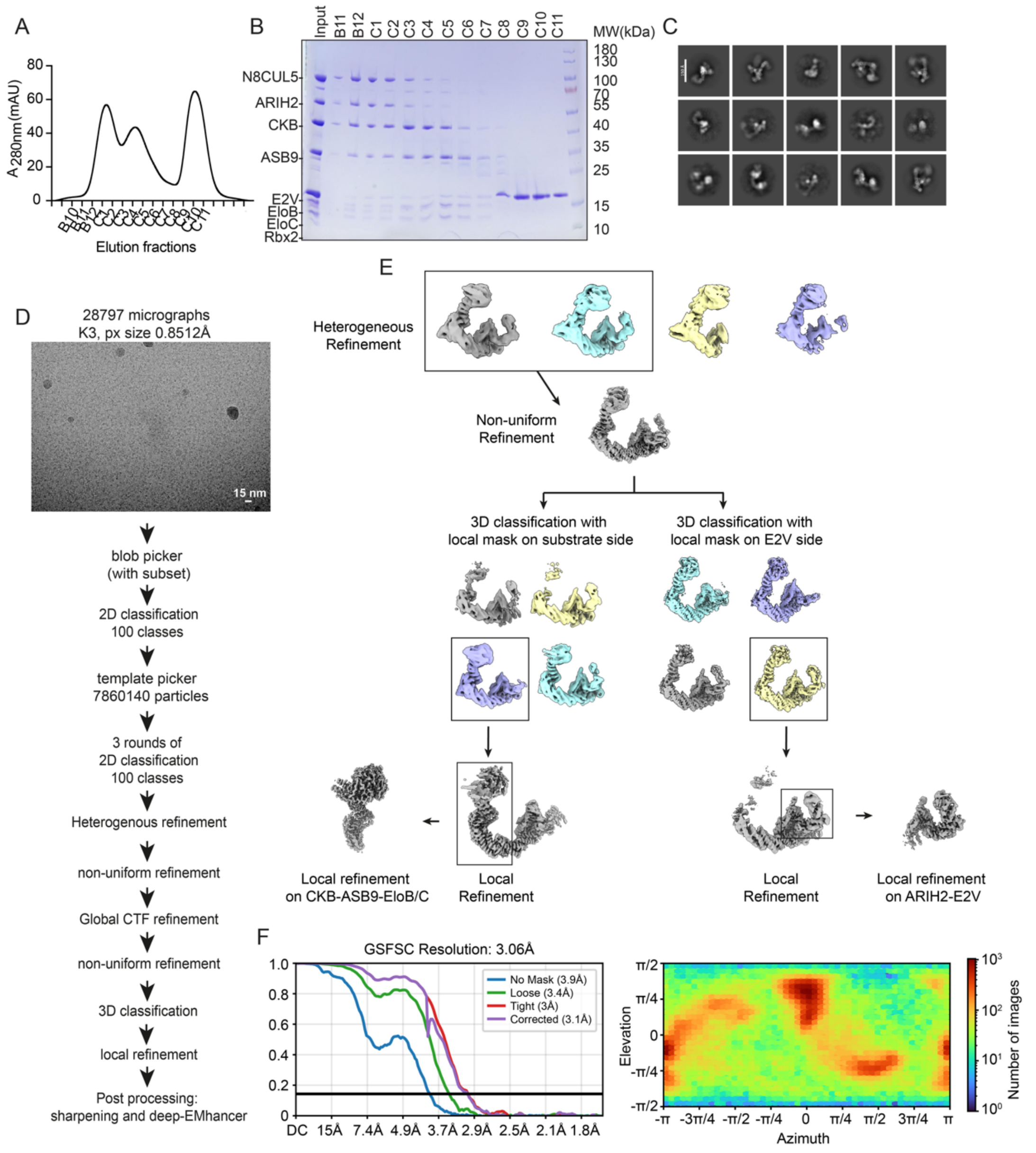

Fig. S6. Structural determination of ABS9-EloB/C-CKB-NEDD8-CUL5-RBX2-ARIH2-L3A2-1by cryo-EM

(A-B) Purification of ABS9-EloB/C-CKB-NEDD8-CUL5-RBX2-ARIH2-L3A2-1 (A) size exclusion chromatogram (B) SDS-PAGE analysis of input and fractions of size exclusion chromatography for cryo-EM. Input, mixture of ABS9-EloB/C-CKB-NEDD8-CUL5-RBX2-ARIH2-L3A2-1 prior to size exclusion chromatography purification.
(C) 2D classification of ABS9-EloB/C-CKB-NEDD8-CUL5-RBX2-ARIH2-L3A2-1 complex.
(D) Cryo-EM processing workflow for ABS9-EloB/C-CKB-NEDD8-CUL5-RBX2-ARIH2-L3A2-1 complex.
(E) Representative maps of heterogenous refinement, non-uniform refinement, 3D classification and local refinement of ABS9-EloB/C-CKB-NEDD8-CUL5-RBX2-ARIH2-L3A2-1.
(F) GSFSC resolution estimation and angular distribution graph of final global refinement of ABS9-EloB/C-CKB-NEDD8-CUL5-RBX2-ARIH2-L3A2-1.

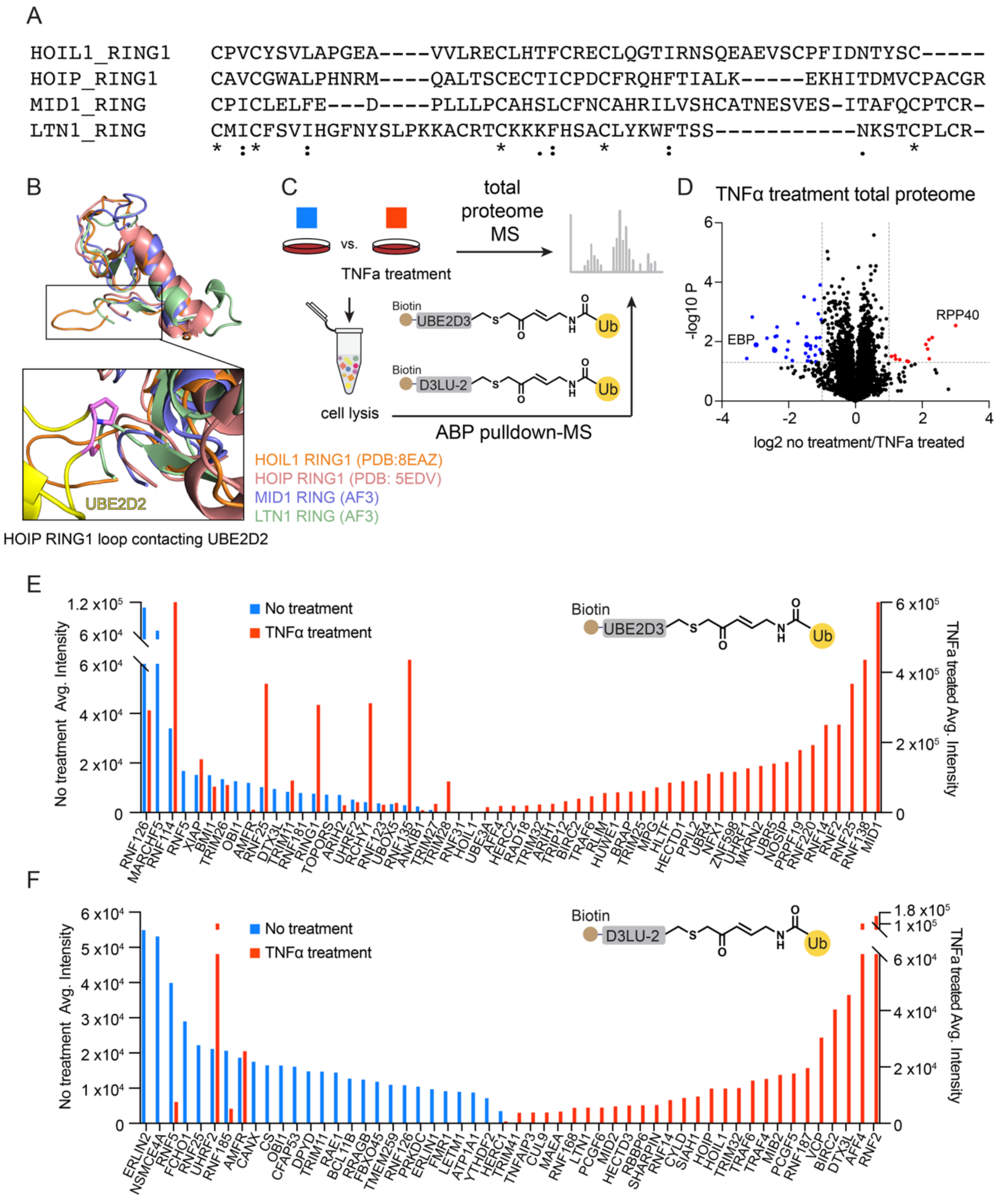

Fig. S7. E3 profiling upon TNFα activation.

(A) Sequence alignment of RING1 domain of HOIL1 and HOIP and RING domain of LTN1 and MID1.
(B) Structural alignment of RING1 domain of HOIL1 (orange, PDB: 8EAZ) and HOIP (peach, PDB:5EDV) and AlphaFold3 predicted RING domain of LTN1 (green) and MID1 (purple).
(C) Scheme for total proteomics or use of E2 and LUBAC E2V derived activity-based probes for cellular RBR E3 ligase profiling with or without treatment of HEK293T cells with TNFα.
(D) Total proteomics of HEK293T with or without TNFα treatment. Proteins that are significantly enriched upon TNFα treatment are shown in blue and significantly downregulated are shown in red. Volcano plot represents mean value of four technical replicates (n=4) for mass spectrometry measurement.

(E-F) Proteins captured by UBE2D3 ABP (C) or D3LU-2 ABP (D) in un-treated (blue) versus TNFα treated (red) HEK293T cells. X-axis shows proteins enriched in either condition. Bar graphs show the mean value of four biological replicates (n=4) for each pulldown mass spectrometry measurement.

Table S1. Cryo-EM data collection, refinement and validation statistics

MAP	NEDD8-CUL5-RBX2-ARIH2~L3A2-1~UB	NEDD8-CUL5-RBX2-ARH2-L3A2-1	ASB9-ELOB/C-CKB
PDB	9SDX	9SDY	-
EMDB	54793	54794	54795
MICROSCOPE	TFS Titan Krios	TFS Titan Krios	TFS Titan Krios
VOLTAGE (KV)	300	300	300
EXPOSURE NAVIGATION	Stage Movement &beamshift	Stage Movement &beamshift	Stage Movement &beamshift
AUTOMATION SOFTWARE	SerialEM	SerialEM	SerialEM
DETECTOR	Gatan K3	Gatan K3	Gatan K3
ENERGY FILTER	10eV	10eV	10eV
NOMINAL MAGNIFICATION	105k	105k	104k
PIXEL SIZE (Å/PIXEL)	0.8512	0.8512	0.8512
EXPOSURE TIME (S), FRAMES	3/40	3/40	3/40
EXPOSURE RATE ($E^-/Å^2\ S^{-1}$)	20	20	20
ELECTRON EXPOSURE ($E^-/Å^2$)	60	60	60
DEFOCUS RANGE (μM)	-1.0 to -2.6	-1.0 to -2.6	-1.0 to -2.6
MICROGRAPHS COLLECTED	18,222	28,797	28,797
SOFTWARE	CryoSPARC v4.2	CryoSPARC v4.2	CryoSPARC v4.2
MICROGRAPHS USED	17,350	25,078	25,078
PARTICLES USED IN REFINEMENT	200,442	191,502	442,136
SYMMETRY	C1	C1	C1
OVERALL RESOLUTION (Å) GOLD-STANDARD FSC (MASKED)	2.97	3.06	2.83
MAP SHARPENING B-FACTOR ($Å^2$)	-83	-88.8	-84
SOFTWARE	Phenix	Phenix	-
NON-HYDROGEN ATOMS	10572	9126	-
PROTEIN RESIDUES	1381	1172	-
AVERAGE B FACTORS ($Å^2$)	129.93	77.67	-
R.M.S. DEVIATIONS			
BOND LENGTH (Å)	0.003	0.003	-
BOND ANGLE (˚)	0.618	0.579	-
RAMACHANDRAN OUTLIERS (%)	0	0	-
MOLPROBITY SCORE	1.82	1.81	-
CLASH SCORE	7.25	7.46	-
POOR ROTAMERS (%)	0	0	-
MODEL VS. MAP FSC =0.143 (MASKED, Å)	1.8	2.1	-

Dataset S1 (separate file). Uncropped gels with molecular weight markers and Coomassie stained fluorescent gels.

Dataset S2 (separate file). Raw data for oligonucleotides design for phage-displayed E2V library construction, ELISA, inhibition kinetics for ARIH2^{ON} and proteomics analysis.

Dataset S3 (separate file). Isothermal titration calorimetry (ITC) and biolayer interferometry (BLI) data for RBR E3 ligases, E2s and E2 variants selected for binding to RBR E3 ligases.

SI References

1. R. Tonikian, Y. Zhang, C. Boone, S. S. Sidhu, Identifying specificity profiles for peptide recognition modules from phage-displayed peptide libraries. Nat Protoc 2, 1368–1386 (2007).
2. S. Kostrhon, et al., CUL5-ARIH2 E3-E3 ubiquitin ligase structure reveals cullin-specific NEDD8 activation. Nat Chem Biol 17, 1075–1083 (2021).
3. D. Horn-Ghetko, et al., Ubiquitin ligation to F-box protein targets by SCF–RBR E3–E3 super-assembly. Nature 590, 671–676 (2021).
4. D. Horn-Ghetko, et al., Noncanonical assembly, neddylation and chimeric cullin–RING/RBR ubiquitylation by the 1.8 MDa CUL9 E3 ligase complex. Nat Struct Mol Biol 31, 1083–1094 (2024).
5. C. M. Pickart, S. Raasi, “Controlled Synthesis of Polyubiquitin Chains” in (2005), pp. 21–36.
6. A. Punjani, J. L. Rubinstein, D. J. Fleet, M. A. Brubaker, cryoSPARC: algorithms for rapid unsupervised cryo-EM structure determination. Nat Methods 14, 290–296 (2017).
7. E. F. Pettersen, et al., UCSF ChimeraX: Structure visualization for researchers, educators, and developers. Protein Science 30, 70–82 (2021).
8. P. Emsley, K. Cowtan, Coot: model-building tools for molecular graphics. Acta Crystallogr D Biol Crystallogr 60, 2126–2132 (2004).
9. D. Liebschner, et al., Macromolecular structure determination using X-rays, neutrons and electrons: recent developments in Phenix. Acta Crystallogr D Struct Biol 75, 861–877 (2019).
10. J. Li, et al., Cullin-RING ligases employ geometrically optimized catalytic partners for substrate targeting. Mol Cell 84, 1304-1320.e16 (2024).
11. J. Liwocha, et al., Mechanism of millisecond Lys48-linked poly-ubiquitin chain formation by cullin-RING ligases. Nat Struct Mol Biol 31, 378–389 (2024).
12. L. A. Hehl, et al., Structural snapshots along K48-linked ubiquitin chain formation by the HECT E3 UBR5. Nat Chem Biol 20, 190–200 (2024).
13. S. A. Maiwald, et al., TRIP12 structures reveal HECT E3 formation of K29 linkages and branched ubiquitin chains. Nat Struct Mol Biol (2025). https://doi.org/10.1038/s41594-025-01561-1.
14. L. T. Henneberg, et al., Activity-based profiling of cullin–RING E3 networks by conformation-specific probes. Nat Chem Biol 19, 1513–1523 (2023).
15. J. R. Wiśniewski, F. Z. Gaugaz, Fast and Sensitive Total Protein and Peptide Assays for Proteomic Analysis. Anal Chem 87, 4110–4116 (2015).
16. P. Skowronek, et al., Rapid and In-Depth Coverage of the (Phospho-) Proteome With Deep Libraries and Optimal Window Design for dia-PASEF. Molecular and Cellular Proteomics 21 (2022).
17. V. Demichev, C. B. Messner, S. I. Vernardis, K. S. Lilley, M. Ralser, DIA-NN: neural networks and interference correction enable deep proteome coverage in high throughput. Nat Methods 17, 41–44 (2020).
18. C. Ammar, J. P. Schessner, S. Willems, A. C. Michaelis, M. Mann, Accurate Label-Free Quantification by directLFQ to Compare Unlimited Numbers of Proteomes. Molecular and Cellular Proteomics 22 (2023).
