# Supplementary material for: E2 variants for probing E3 ubiquitin ligase activities
Source: Proc Natl Acad Sci U S A. 2026 Jan 2;123(1):e2524899122. doi: 10.1073/pnas.2524899122 (PMC12773759; doi:10.1073/pnas.2524899122)

Fig.2A

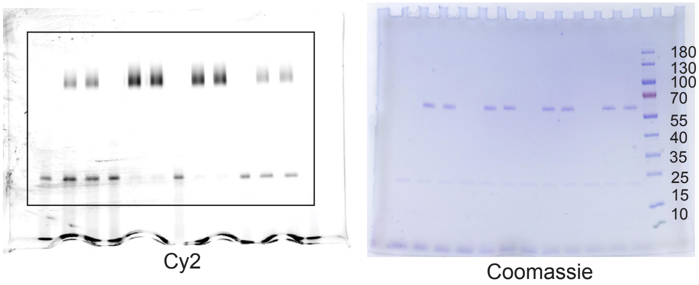

Fig.2C

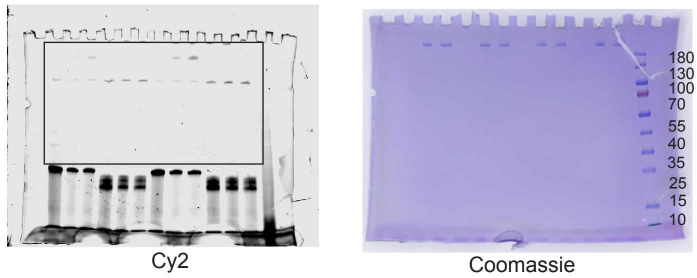

Fig.2D

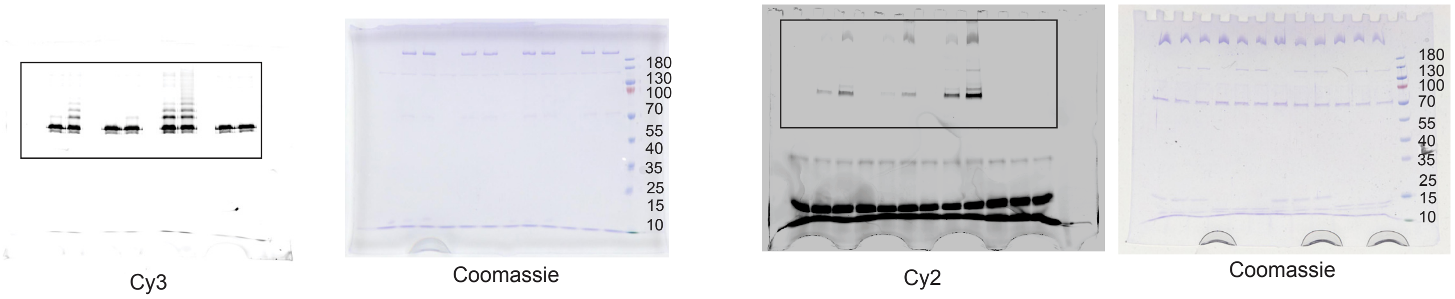

Fig.3B

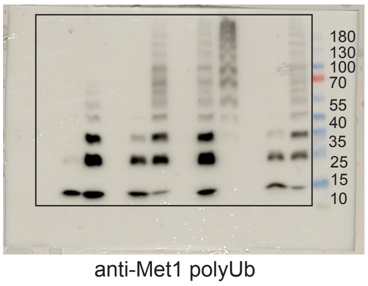

Fig.3C

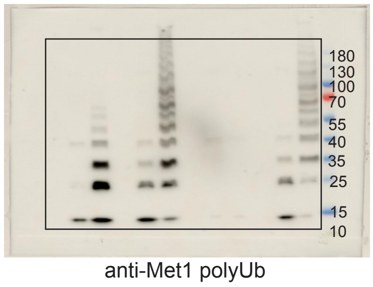

Fig.3D

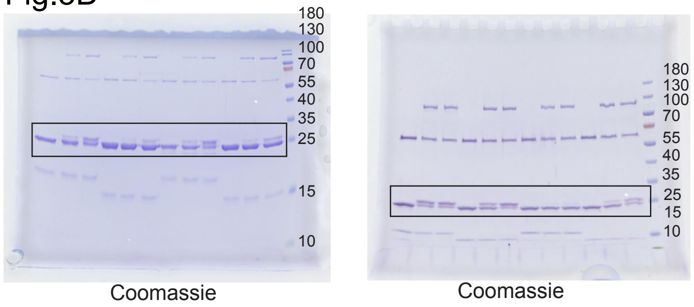

Fig.4B

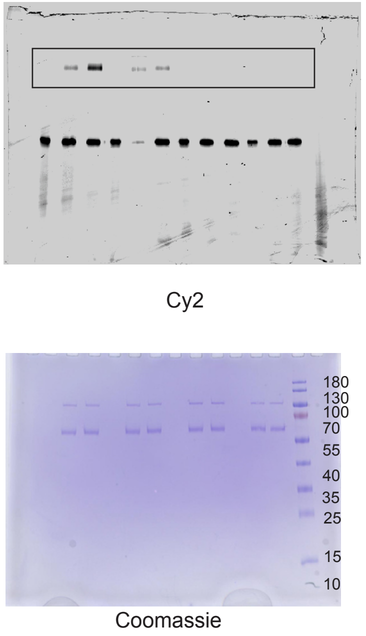

Fig.4D

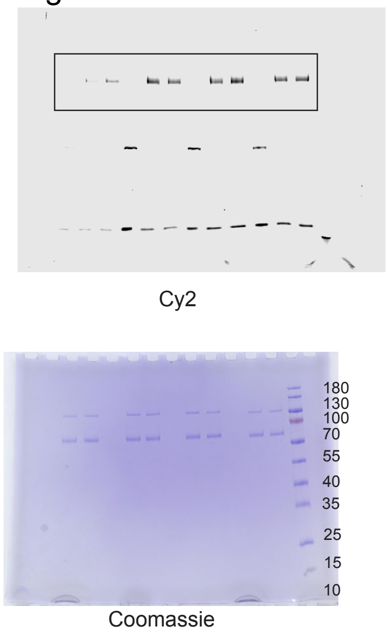

Fig.4E

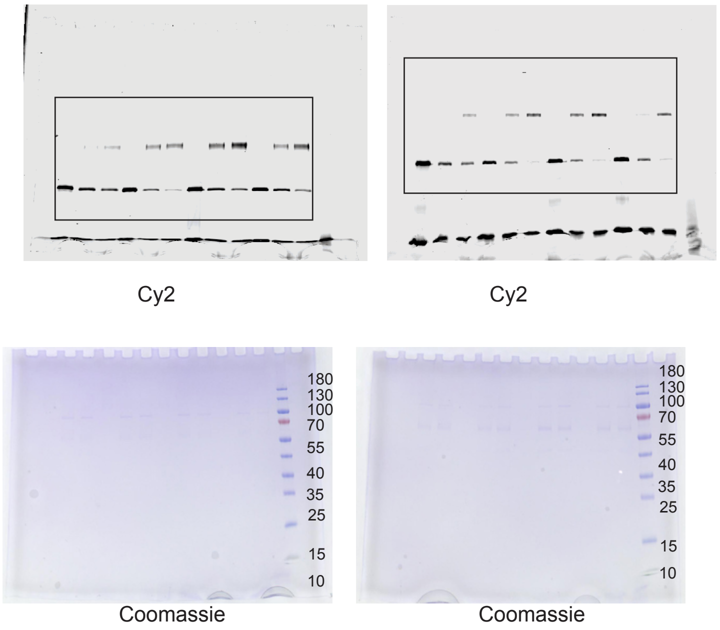

Fig.S2A

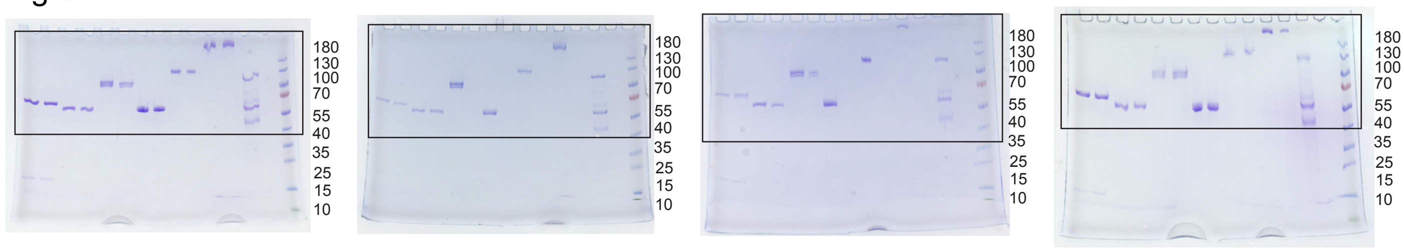

Fig.S2B

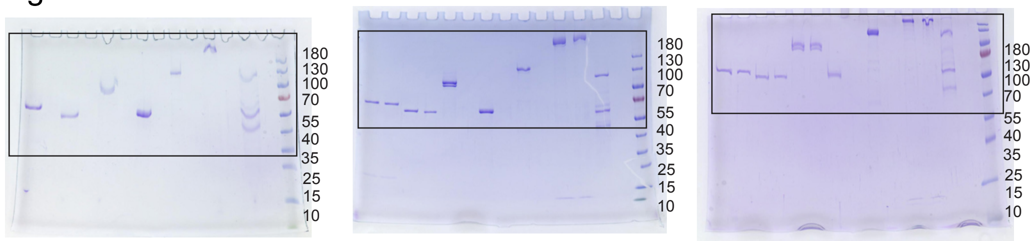

Fig.S2C

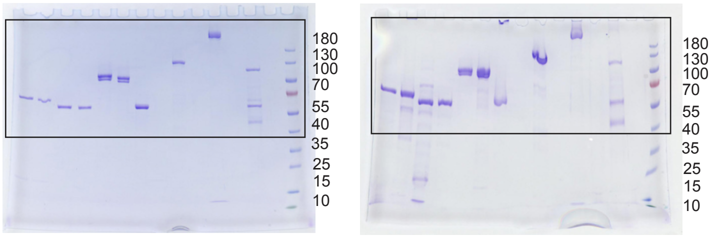

Fig.S2F

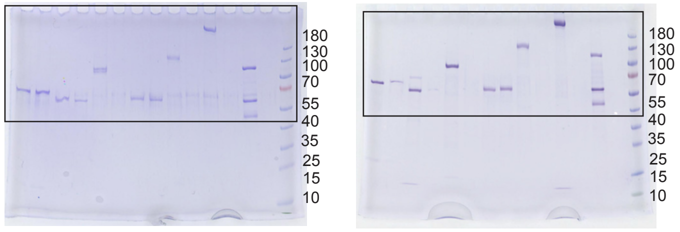

Fig.S2E

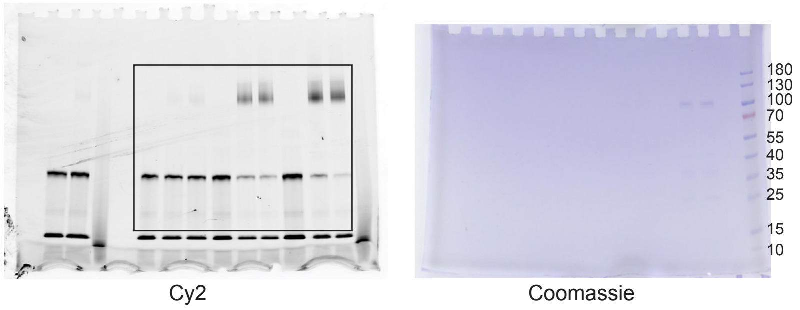

Fig.S2G

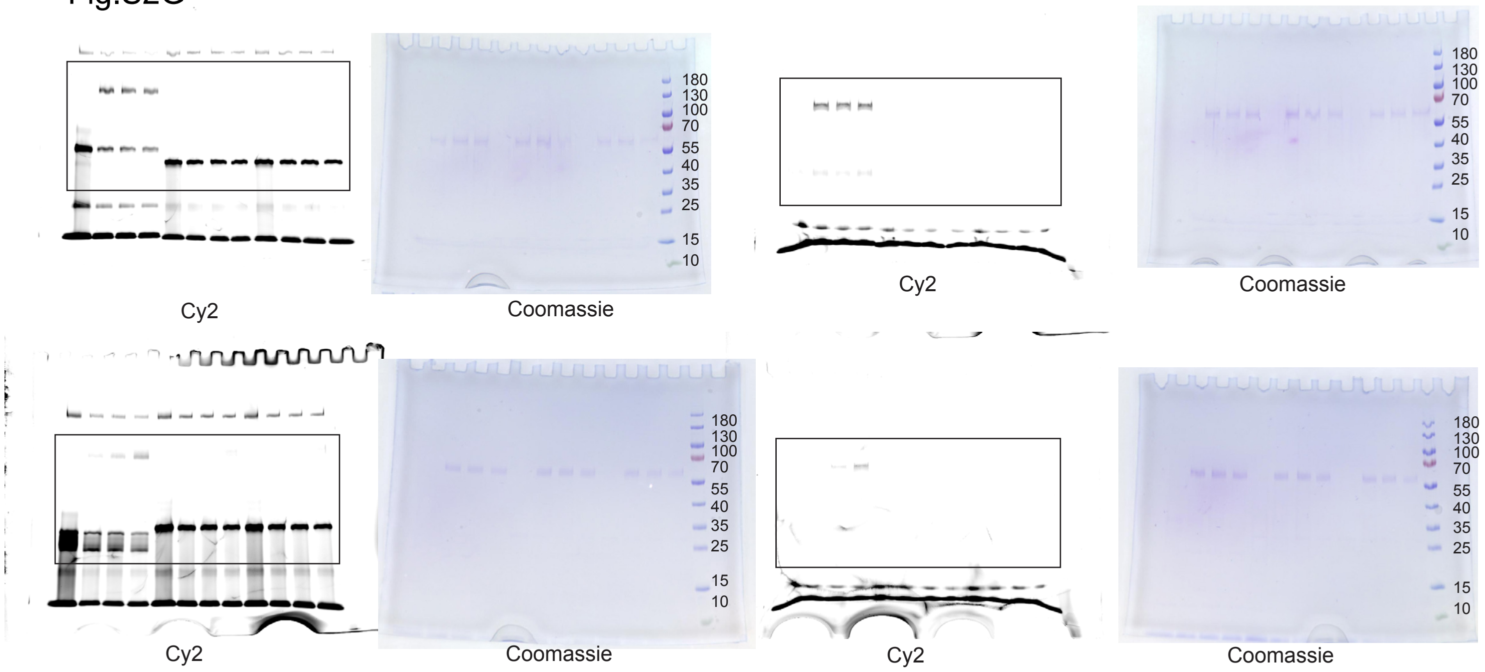

Fig.S2G

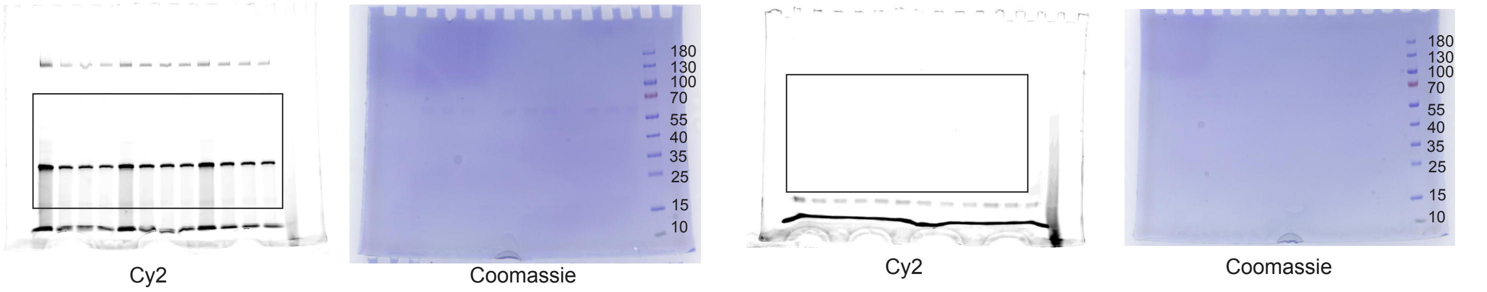

Fig.S2I

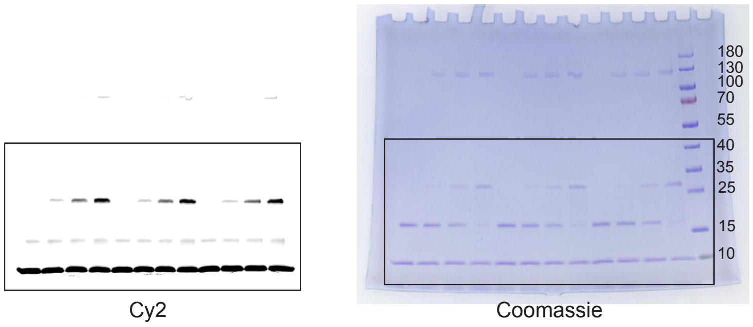

Fig.S2J

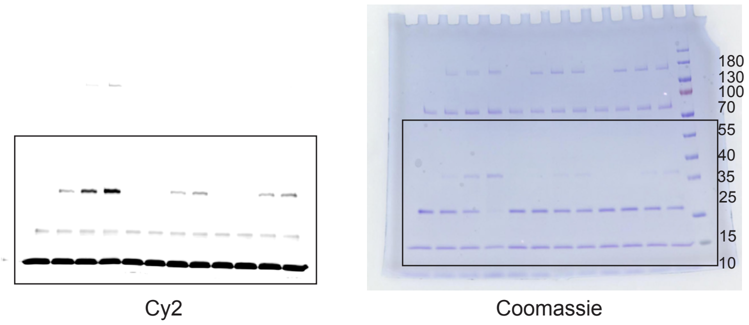

Fig.S2K

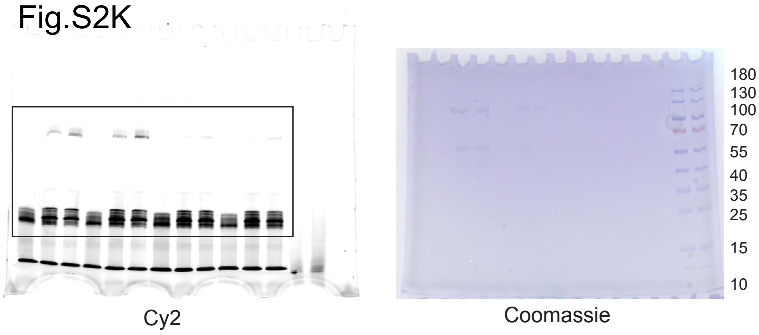

Fig.S2L

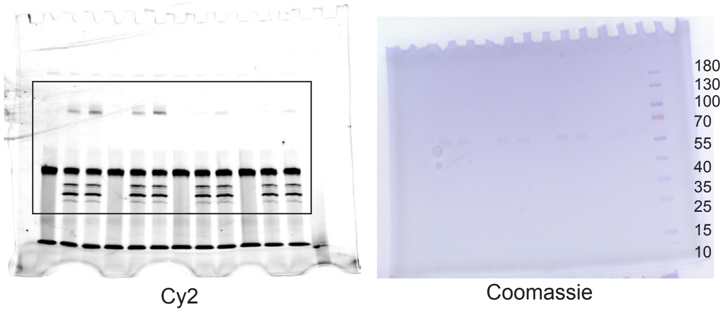

Fig.S3A

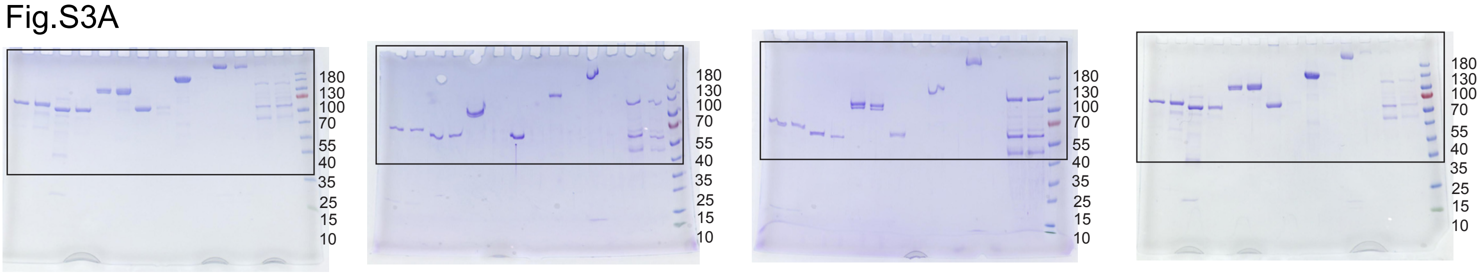

Fig.S3B

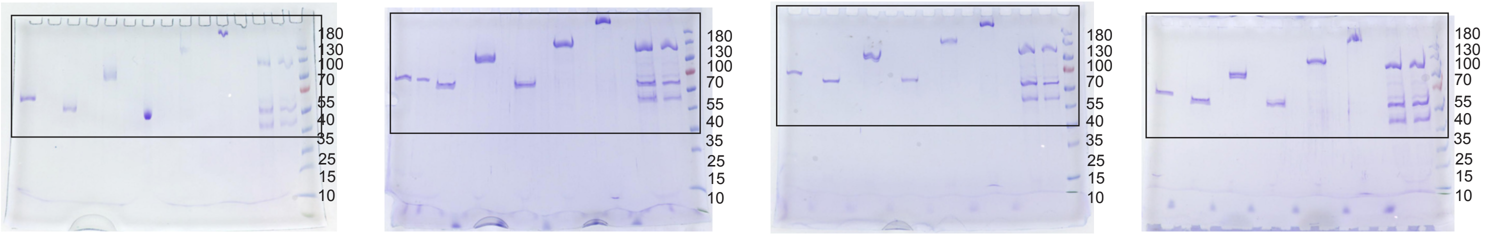

Fig.S3C

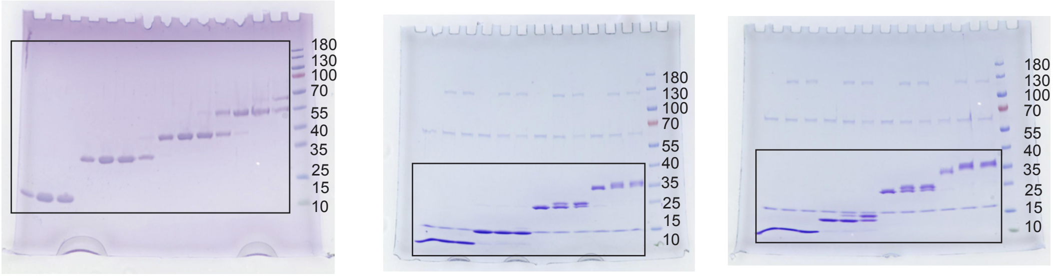

Fig.S3D

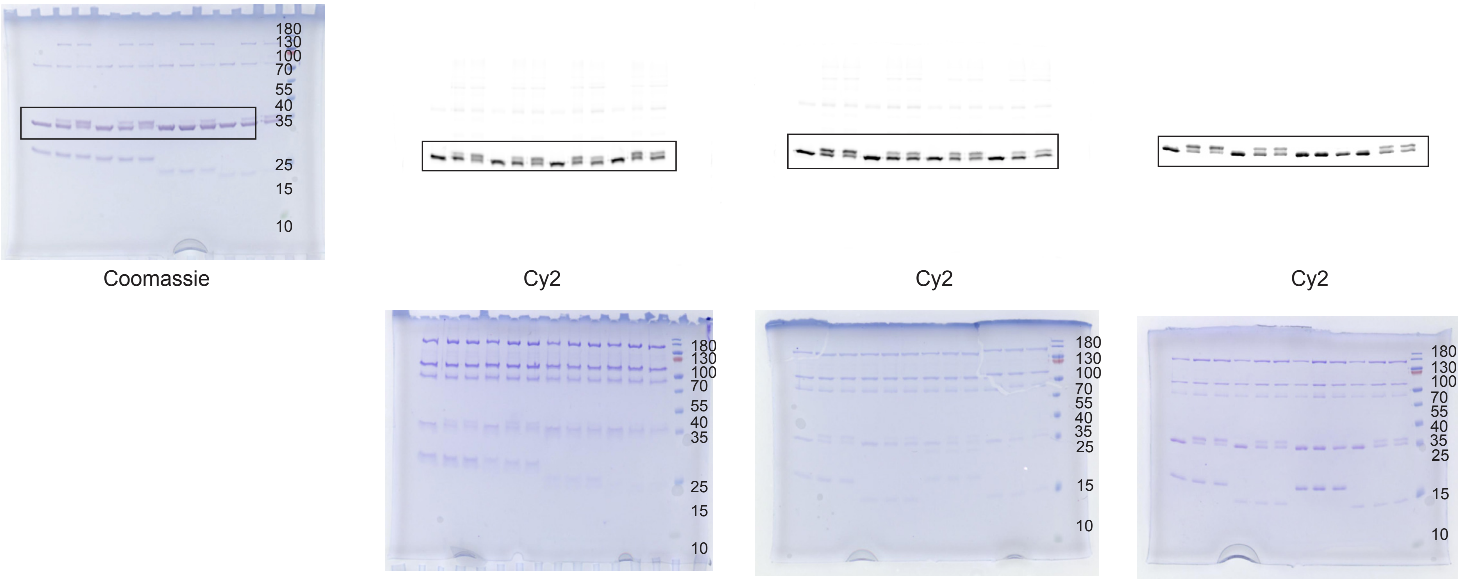

Coomassie Cy2 Cy2 Cy2

Coomassie Coomassie Coomassie

Fig.S3E

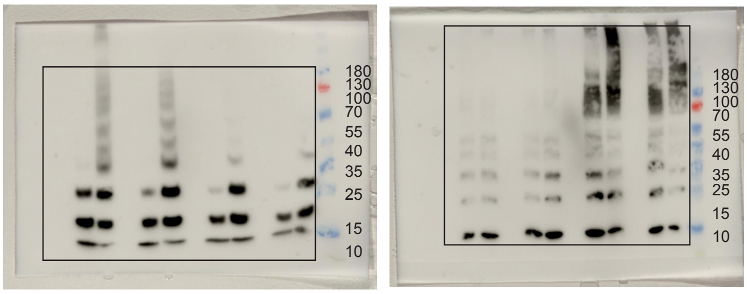

anti-Met1 polyUb anti-Met1 polyUb

Fig.3F

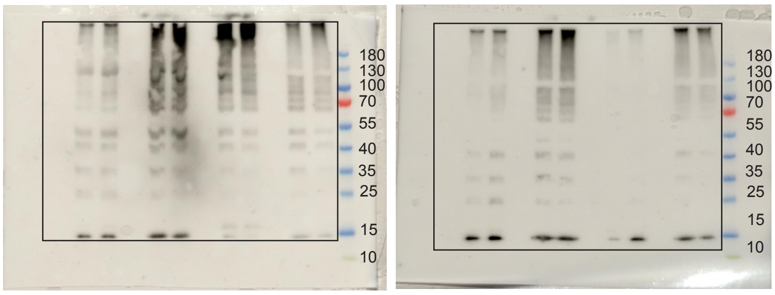

anti-Met1 polyUb anti-Met1 polyUb

Fig.S4A

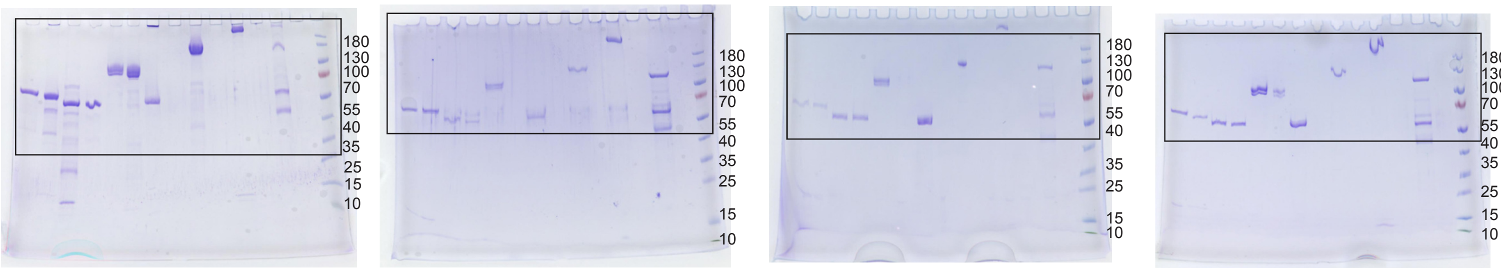

Fig.S4A

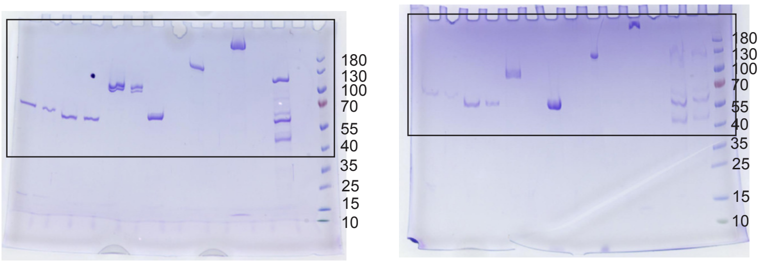

Fig.S4D

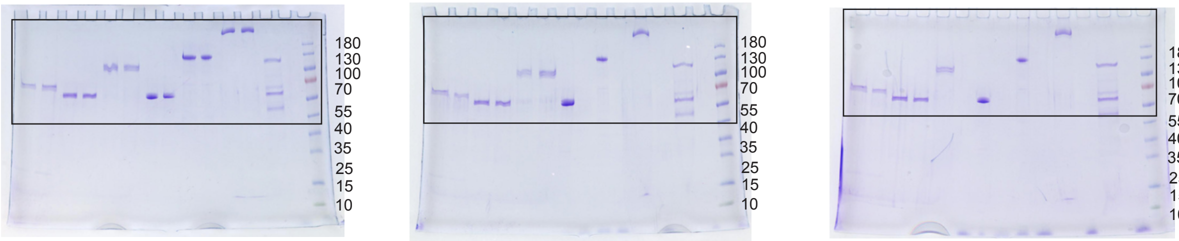

Fig.S4E

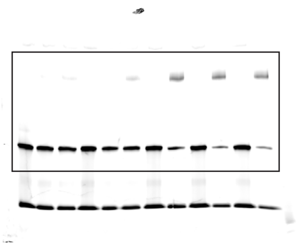

Cy2

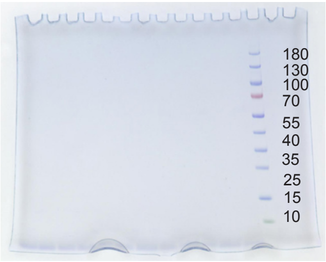

Coomassie

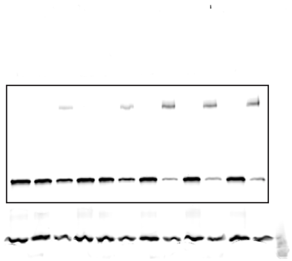

Cy2

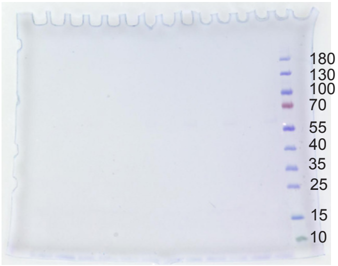

Coomassie

Fig.S5C

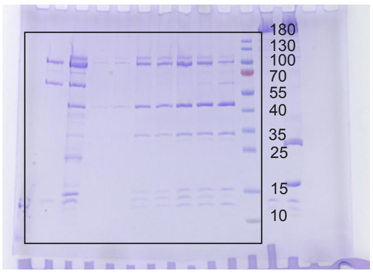

Fig.S6B

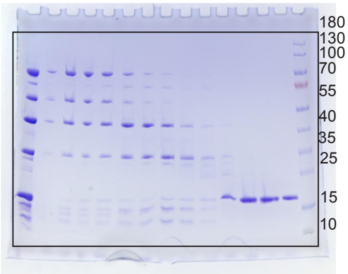

Kinetics gels for quantification (Cy2)

0 μM L3A2-1≠

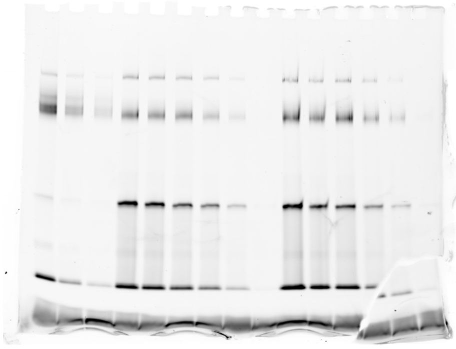

0.05 μM L3A2-1≠

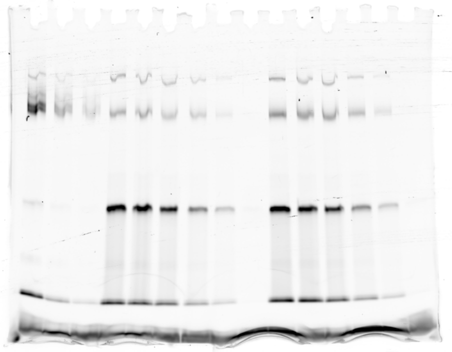

0.1 μM L3A2-1≠

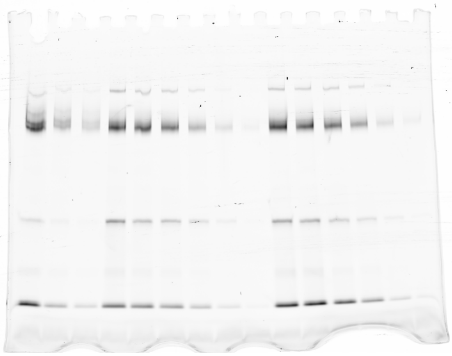

0.2 μM L3A2-1≠

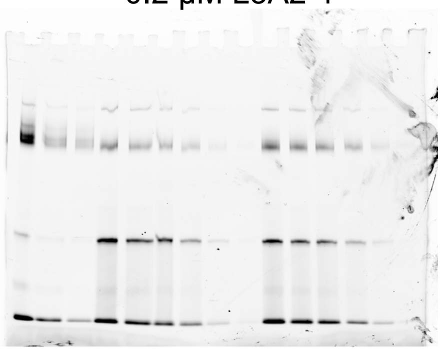

0.3 μM L3A2-1≠

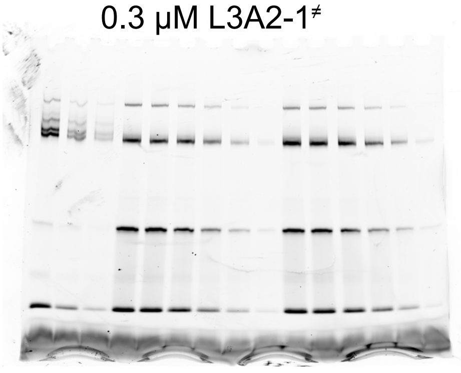

0.4 μM L3A2-1≠

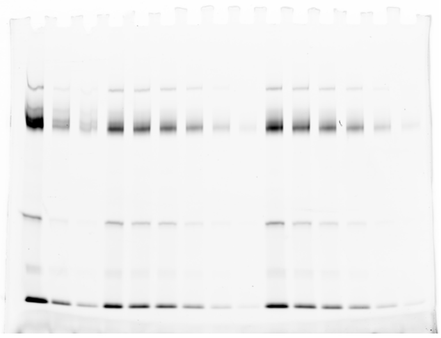

0.5 μM L3A2-1≠

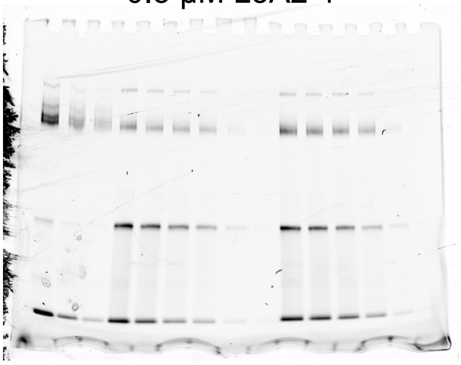

Supplement: Supplementary file 2 — Dataset S01 (PDF) [file pnas.2524899122.sd01.pdf]
