# Supplementary material for: E2 variants for probing E3 ubiquitin ligase activities
Source: Proc Natl Acad Sci U S A. 2026 Jan 2;123(1):e2524899122. doi: 10.1073/pnas.2524899122 (PMC12773759; doi:10.1073/pnas.2524899122)
Supplement: Supplementary file 4 — Dataset S03 (PDF) [file pnas.2524899122.sd03.pdf]

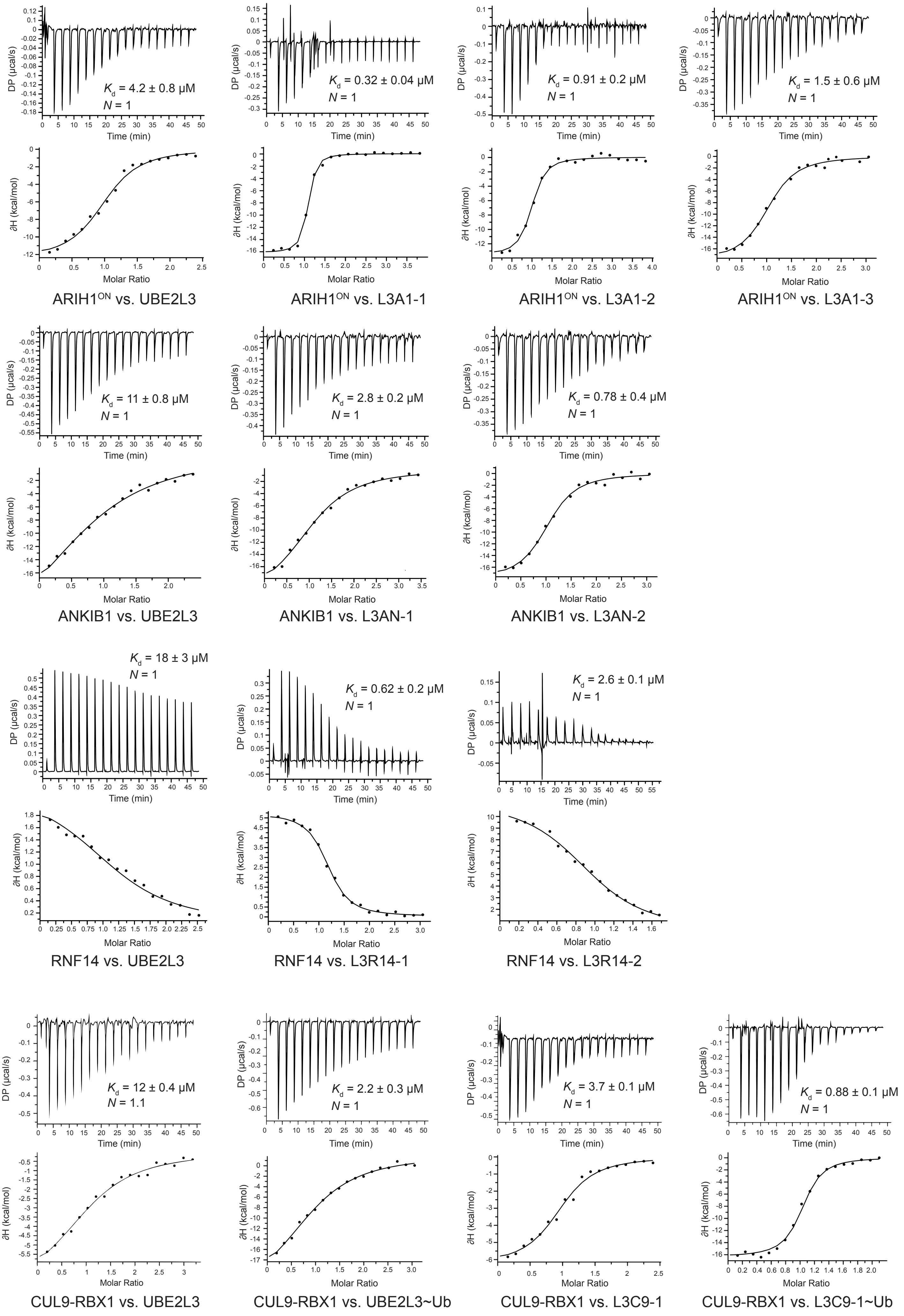

K_d = 4.2 ± 0.8 μM
N = 1
ARIH1^{ON} vs. UBE2L3
K_d = 0.32 ± 0.04 μM
N = 1
ARIH1^{ON} vs. L3A1-1
K_d = 0.91 ± 0.2 μM
N = 1
ARIH1^{ON} vs. L3A1-2
K_d = 1.5 ± 0.6 μM
N = 1
ARIH1^{ON} vs. L3A1-3
K_d = 11 ± 0.8 μM
N = 1
ANKIB1 vs. UBE2L3
K_d = 2.8 ± 0.2 μM
N = 1
ANKIB1 vs. L3AN-1
K_d = 0.78 ± 0.4 μM
N = 1
ANKIB1 vs. L3AN-2
K_d = 18 ± 3 μM
N = 1
RNF14 vs. UBE2L3
K_d = 0.62 ± 0.2 μM
N = 1
RNF14 vs. L3R14-1
K_d = 2.6 ± 0.1 μM
N = 1
RNF14 vs. L3R14-2
K_d = 12 ± 0.4 μM
N = 1.1
CUL9-RBX1 vs. UBE2L3
K_d = 2.2 ± 0.3 μM
N = 1
CUL9-RBX1 vs. UBE2L3~Ub
K_d = 3.7 ± 0.1 μM
N = 1
CUL9-RBX1 vs. L3C9-1
K_d = 0.88 ± 0.1 μM
N = 1
CUL9-RBX1 vs. L3C9-1~Ub
DP (μcal/s)
Time (min)
∂H (kcal/mol)
Molar Ratio

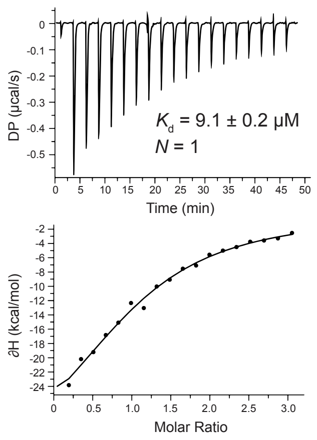

CUL9-RBX1 vs. UBE2D3~Ub

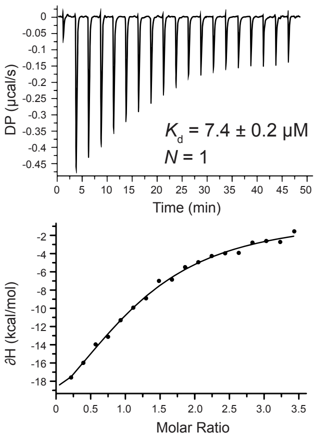

CUL9-RBX1 vs. D3C9-1

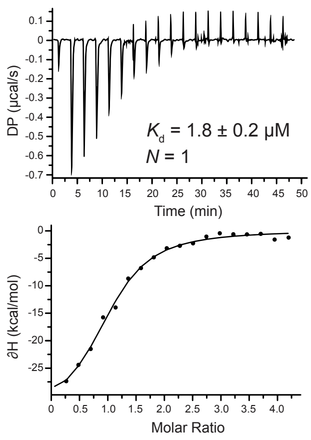

CUL9-RBX1 vs. D3C9-1~Ub

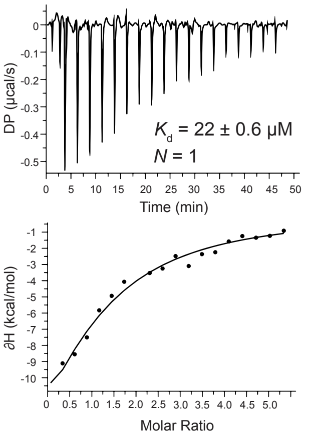

LUBAC vs. UBE2L3

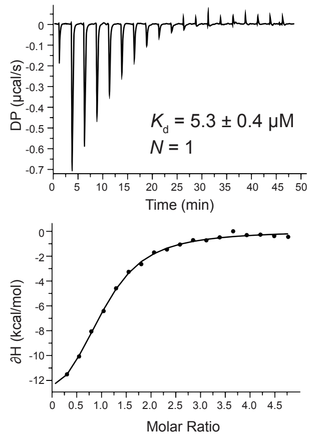

LUBAC vs. UBE2L3~Ub

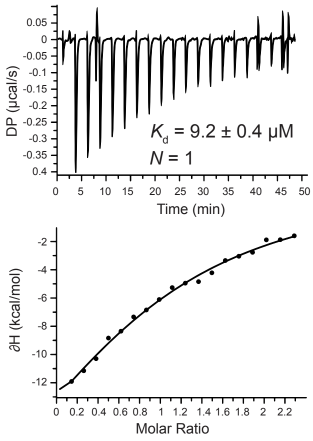

LUBAC vs. UBE2D3

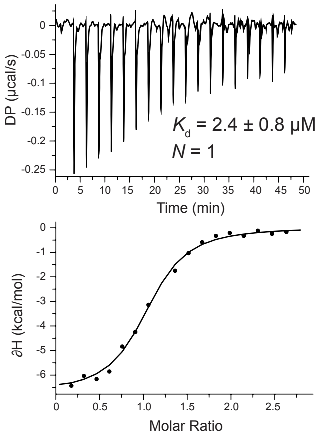

LUBAC vs. UBE2D3~Ub

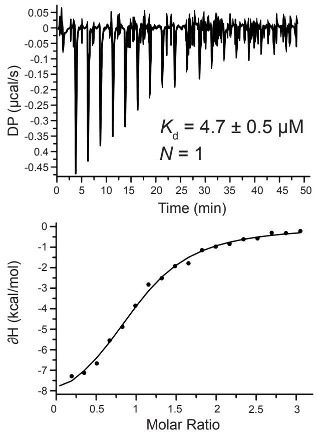

LUBAC vs. L3LU-1

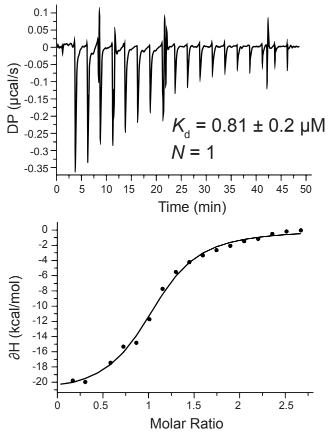

LUBAC vs. L3LU-1~Ub

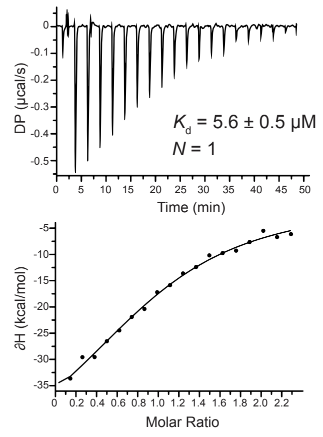

LUBAC vs. L3LU-2

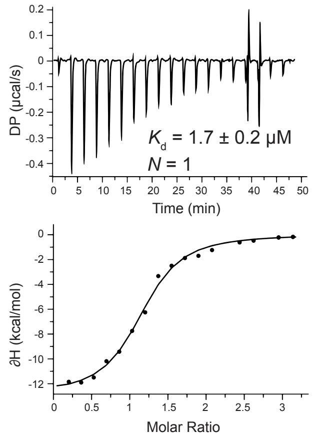

LUBAC vs. L3LU-2~Ub

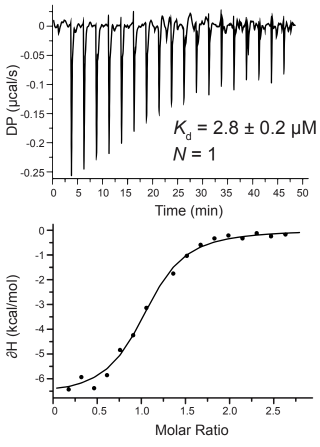

LUBAC vs. D3LU-1

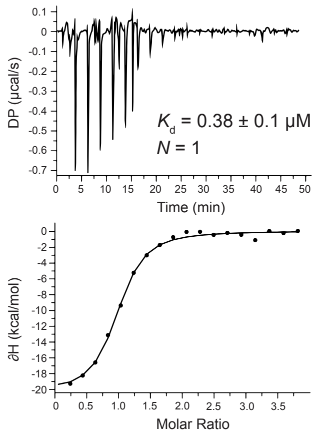

LUBAC vs. D3LU-1~Ub

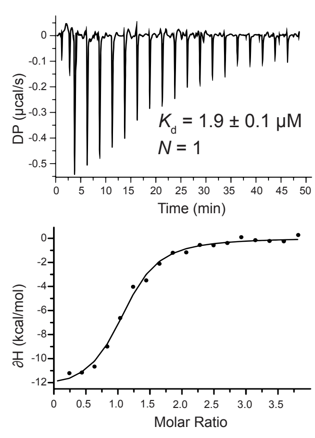

LUBAC vs. D3LU-2

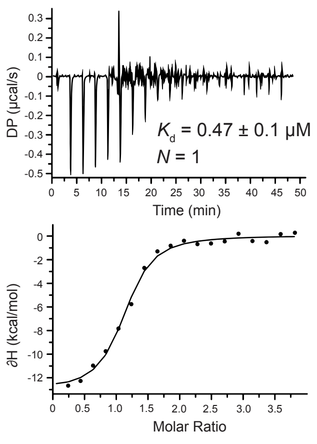

LUBAC vs. D3LU-2~Ub

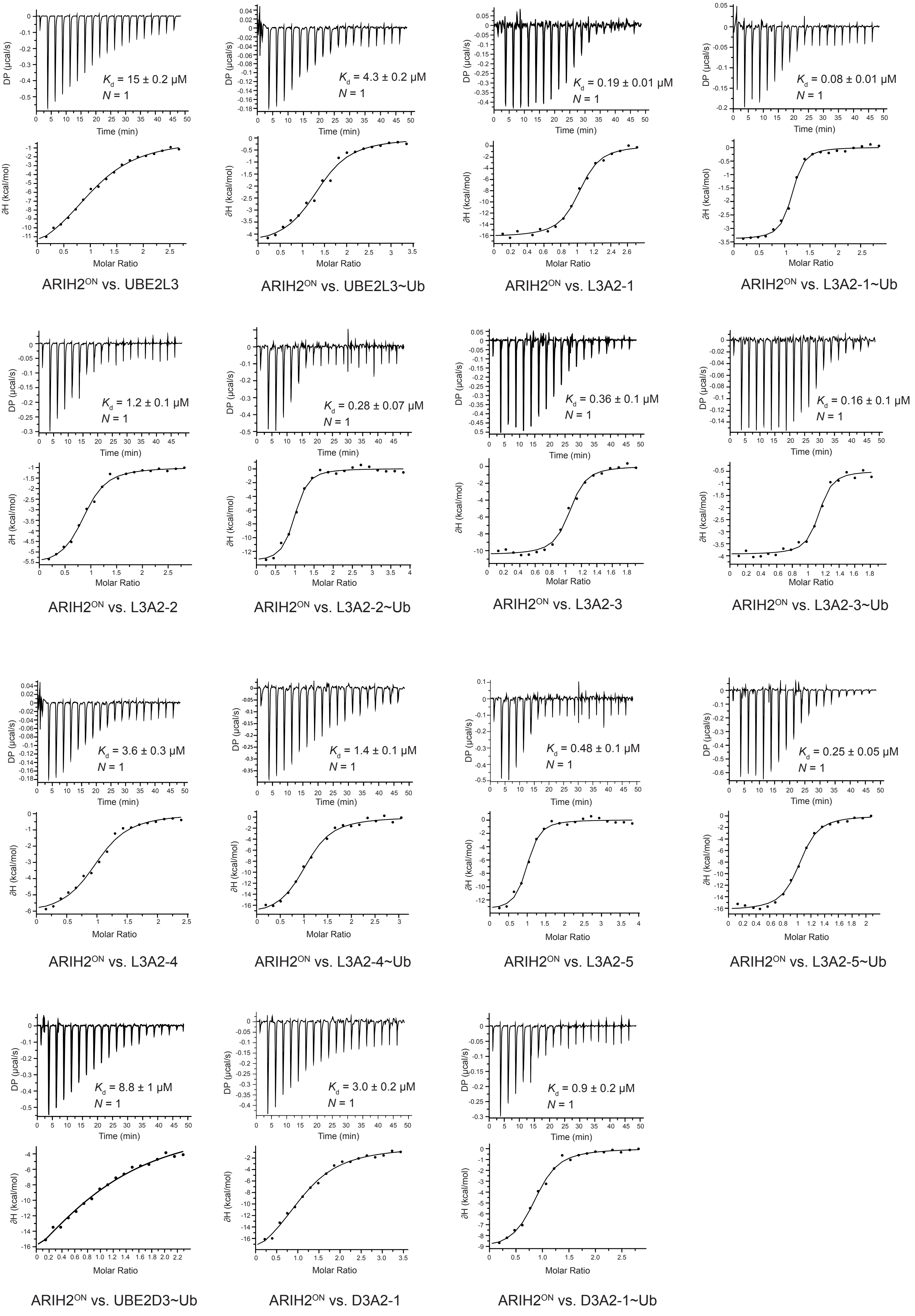

DP (µcal/s)
Time (min)
∂H (kcal/mol)
Molar Ratio
K_d = 15 ± 0.2 µM
N = 1
ARIH2^{ON} vs. UBE2L3
K_d = 4.3 ± 0.2 µM
N = 1
ARIH2^{ON} vs. UBE2L3~Ub
K_d = 0.19 ± 0.01 µM
N = 1
ARIH2^{ON} vs. L3A2-1
K_d = 0.08 ± 0.01 µM
N = 1
ARIH2^{ON} vs. L3A2-1~Ub
K_d = 1.2 ± 0.1 µM
N = 1
ARIH2^{ON} vs. L3A2-2
K_d = 0.28 ± 0.07 µM
N = 1
ARIH2^{ON} vs. L3A2-2~Ub
K_d = 0.36 ± 0.1 µM
N = 1
ARIH2^{ON} vs. L3A2-3
K_d = 0.16 ± 0.1 µM
N = 1
ARIH2^{ON} vs. L3A2-3~Ub
K_d = 3.6 ± 0.3 µM
N = 1
ARIH2^{ON} vs. L3A2-4
K_d = 1.4 ± 0.1 µM
N = 1
ARIH2^{ON} vs. L3A2-4~Ub
K_d = 0.48 ± 0.1 µM
N = 1
ARIH2^{ON} vs. L3A2-5
K_d = 0.25 ± 0.05 µM
N = 1
ARIH2^{ON} vs. L3A2-5~Ub
K_d = 8.8 ± 1 µM
N = 1
ARIH2^{ON} vs. UBE2D3~Ub
K_d = 3.0 ± 0.2 µM
N = 1
ARIH2^{ON} vs. D3A2-1
K_d = 0.9 ± 0.2 µM
N = 1
ARIH2^{ON} vs. D3A2-1~Ub

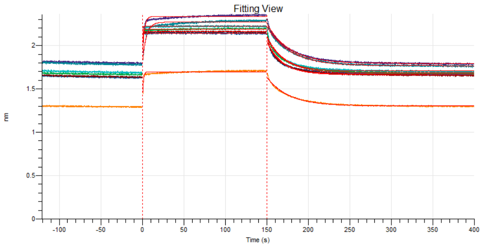

GS-UBE2L3 Kd = 1.4 μM

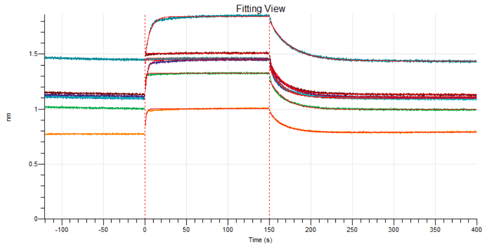

UBE2L3 Kd = 2.8 μM

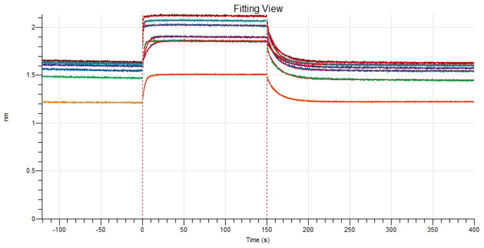

GSM-L3A2-1 Kd = 65.0 nM

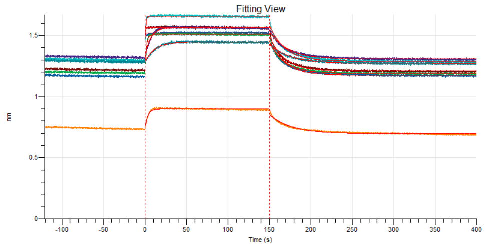

M-L3A2-1 Kd = 68.9 nM

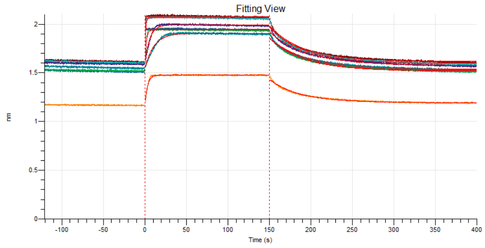

L3A2-1 Kd = 69.1 nM
